# Fe_2_O_3_/Bi_2_WO_6_ hierarchical microspheres for visible-light-driven degradation of *p*-nitrophenol: performance, mechanism, and green metrics

**DOI:** 10.1039/d6ra06864b

**Published:** 2026-09-09

**Authors:** Manmeet Kaur, Amit Dhir, Shilpi Verma

**Affiliations:** a Department of Energy and Environment, Thapar Institute of Engineering & Technology Patiala-147004 Punjab India amit.dhir@thapar.edu shilpi.verma@thapar.edu

## Abstract

Bismuth tungstate (Bi_2_WO_6_), an Aurivillius-phase visible-light semiconductor, has a wide band gap and rapid charge-carrier recombination that significantly affects its photocatalytic efficiency. To overcome these intrinsic limitations, Fe_2_O_3_/Bi_2_WO_6_ composites were synthesised *via* a facile single-step hydrothermal technique with Fe_2_O_3_ loadings of 1, 3, and 5 wt%. XRD confirmed that the orthorhombic Bi_2_WO_6_ phase was retained without segregated Fe_2_O_3_, indicating that the incorporation of Fe into the lattice was successful. Raman spectroscopy proved that the WO_6_ octahedral framework was distorted by Fe, as evidenced by the broadening of the W–O stretching (∼800 cm^−1^) and O–W–O bending (∼308 cm^−1^) modes. FEG-SEM revealed morphological modifications in the hierarchical nanosheet assemblies, and UV-DRS showed a band-gap narrowing from 3.1 eV to 2.9 eV, leading to extended visible-light absorption. BET analysis confirmed comparable mesopores. PL spectroscopy, on the other hand, demonstrated the lowest charge-carrier recombination rate for the 1% Fe_2_O_3_/Bi_2_WO_6_ composite, consistent with its superior photocatalytic performance. XPS investigations further confirmed the slight distortion in the Bi–O coordination environment induced by the inclusion of Fe. Under optimized conditions (catalyst dose: 250 mg L^−1^, pH 7, and PNP: 20 mg L^−1^), the 1% Fe_2_O_3_/Bi_2_WO_6_ composite achieved 84.5% PNP removal under visible-light irradiation, following pseudo-first-order kinetics (*k*_app_ = 0.0093 min^−1^). The photocatalyst exhibited good stability and reusability. In addition, the PNP degradation pathway was proposed based on HR-MS-identified intermediates and supported by TOC analysis and reactive-species detection. The process sustainability was further assessed based on green chemistry metrics, including water intensity, mass intensity, reaction mass intensity, E-factor, and energy consumption, demonstrating favorable environmental credentials relative to conventional remediation methods. These findings identify 1% Fe_2_O_3_/Bi_2_WO_6_ as an efficient and sustainable visible-light photocatalyst for the remediation of recalcitrant phenolic wastewater.

## Abstract 2

Hierarchical Fe_2_O_3_/Bi_2_WO_6_ microspheres improve charge separation, attaining around 84% visible-light degradation of *p*-nitrophenol with favourable green metrics.

## Graphical abstract

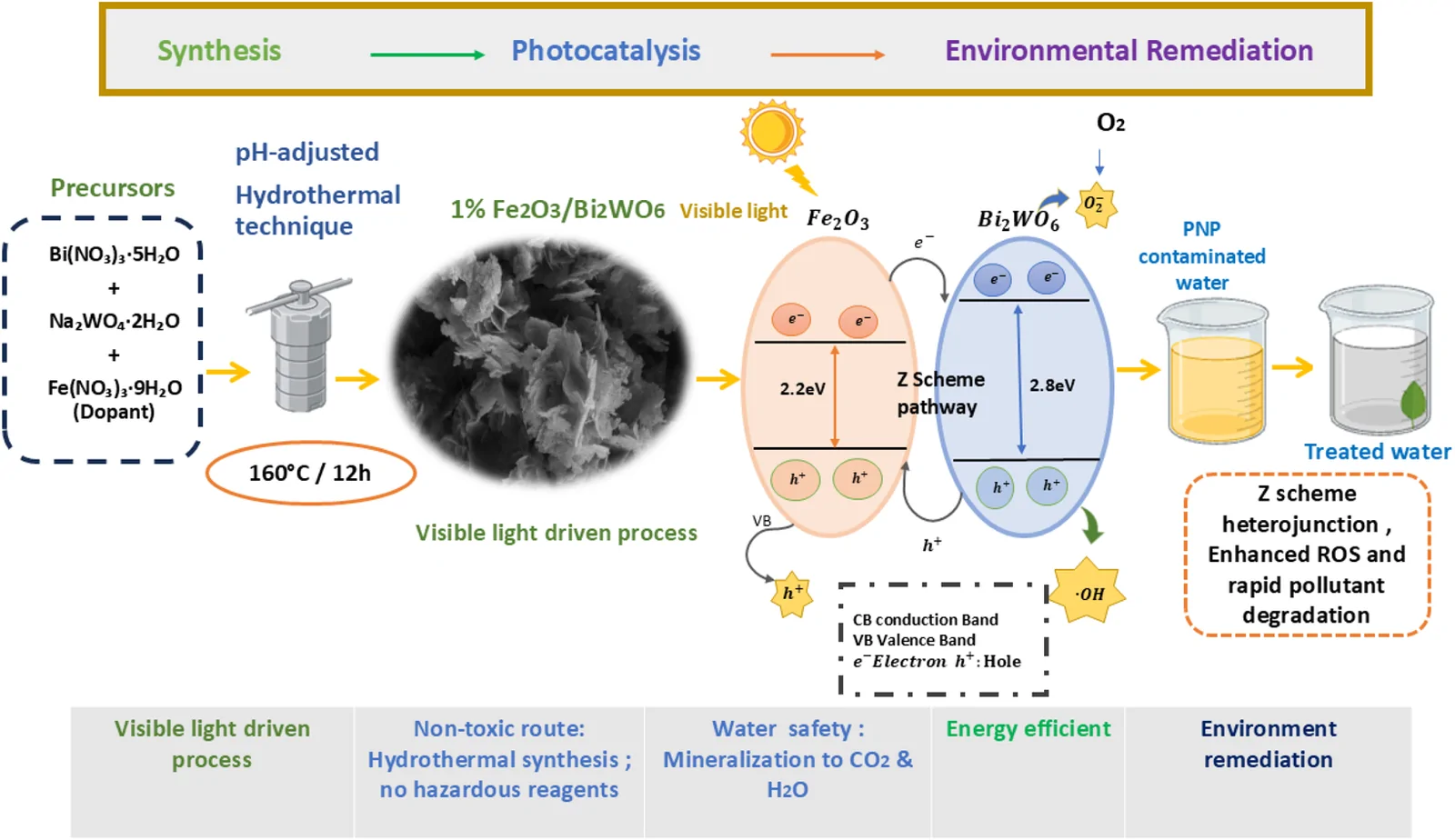

## 1. Introduction

The rapid pace of industrialization and urbanization has intensified the discharge of toxic organic micropollutants into aquatic streams, posing a significant threat to human health and environmental integrity.^1^ Amongst these contaminants, the class of phenolic compounds, particularly *p*-nitrophenol (PNP), commonly known as 4-nitrophenol, has emerged as a priority pollutant of emerging concern.^2^ It functions as an essential intermediary in the pharmaceutical, pesticide, herbicide, dye, explosive and chemical industries, and it enters aquatic systems through industrial effluents and agricultural runoff.^3^ The nitroaromatic group provides exceptional stability in chemical terms, and the strong electron-withdrawing character of the NO_2_ moiety severely impedes biodegradation, enabling PNP to persist and bioaccumulate in aquatic organisms.^4^ The US EPA has classified PNP as a priority pollutant, with permissible concentration limits ranging from 0.01 to 2.0 µg L^−1^. It is known to cause damage to the central nervous system, liver, and kidneys in human beings.^5^ Acute exposure effects include dermatitis, headaches, nausea, eye irritation, and cyanosis. Chronic exposure can lead to bronchitis, coughing, shortness of breath, and reduced ATP production, along with renal failure and other serious health impacts. PNP shows moderate acute toxicity through methemoglobinemia and organ damage. It has also shown weak mutagenicity *in vitro* but insufficient evidence for *in vivo* carcinogenicity.^6^

The efficient removal of PNP from water is a crucial and imperative issue. Although a wide array of conventional treatment technologies, such as adsorption, photocatalytic oxidation/reduction, and electrolytic reduction, exist,^7^ their actual implementation is hampered by their limitations. Adsorption is a non-destructive process that simply transfers PNP onto a solid matrix and produces a contaminated adsorbent that must be disposed of or regenerated at high expense.^8^ PNP's intrinsic toxicity to microbial consortia impedes biological treatment, resulting in lengthy acclimation times and inconsistent efficacy in actual wastewater. Although ozonation results in significant elimination, it requires constant, energy-intensive ozone production and frequently results in only partial mineralization, resulting in hazardous intermediates like hydroquinone and nitrophenolic byproducts. In membrane filtration processes, PNP is concentrated rather than degraded, which causes fouling and produces a reject stream having very high concentrations of PNP, as secondary waste.^9^

The implementation of individual advanced oxidation processes (AOPs) or combinations of two or more AOPs has been demonstrated to produce higher levels of free radicals and improve oxidation efficiency.^10,11^ Most AOPs, including photocatalysis, depend on the production of highly reactive species, such as ˙OH, SO_4_˙, hydrated electrons and^1^ O_2_, which can efficiently oxidize and degrade a diverse array of organic pollutants.^12,13^

In photocatalytic systems, metal tungstate (MWO_4_) has gained significant importance due to its advantageous optical properties and chemical durability.^14,15^ It constitutes two primary crystallographic modifications: the wolframite-type monoclinic structure and the scheelite-type tetragonal structure.^16^ This property enables morphology tuning and allows stable activity to be maintained with resistance to light corrosion. Recent studies have extensively examined bismuth-based catalysts, particularly bismuth tungstate (Bi_2_WO_6_), for photocatalysis.^17,18^ These catalysts are regarded as promising due to their distinctive Aurivillius phase structure, abundance, cost-effectiveness, non-toxicity, optimal energy band gap, strong visible-light absorption, and superior oxidation capability. With a low band gap and an n-type perovskite layered structure, Bi_2_WO_6_ exhibits robust photocatalytic activity as a metal tungstate. However, its widespread use has been constrained by its shorter charge-carrier lifetime and enhanced photoexcited electron–hole recombination. Bi_2_WO_6_ demonstrates photocatalytic activity in both the visible and ultraviolet spectrums.^19^ The formation of Fe-doped Bi_2_WO_6_ hierarchical microsphere structures by coupling different semiconductors or metals to the photocatalyst significantly overcomes these constraints.^11^

Coupling Bi_2_WO_6_ with iron oxide (Fe_2_O_3_) to form Fe-doped Bi_2_WO_6_ hierarchical microspheres seems to be a positive approach.^20,21^ Fe_2_O_3_ is an n-type semiconductor with a narrow band gap (∼2.1–2.2 eV), excellent visible-light absorption, chemical stability, low cost, and environmentally benign nature. The construction of Fe_2_O_3_/Bi_2_WO_6_ creates a staggered band alignment that promotes spatial separation of photogenerated charge carriers across the interface, substantially suppressing electron–hole recombination, which is a critical bottleneck in single-component photocatalysts.^22^ Furthermore, the Fe^3+^/Fe^2+^ redox cycle facilitated by Fe_2_O_3_ can act as an electron mediator, enabling Fenton-like reactions that generate additional hydroxyl radicals –(˙OH) under visible-light irradiation and further enhancing oxidative degradation efficiency.^23^ A comprehensive review of the literature has demonstrated that by extending light absorption and promoting photogenerated charge separation, Fe-modified Bi_2_WO_6_ and Fe_2_O_3_/Bi_2_WO_6_ composites can enhance the visible-light photocatalytic performance of pristine Bi_2_WO_6_. Nguyen *et al.*^24^ synthesised Fe-doped Bi_2_WO_6_ photocatalysts that removed 70.6% of cefalexin (CFX) and degraded Rhodamine B (RhB) by 97.7% after 180 minutes of light exposure. El Aouni *et al.*^25^ synthesized Bi_2_WO_6_ for visible-light-driven degradation of RhB and methyl orange (MO), obtaining degradation efficiencies of 97% and 92%, respectively, in 120 minutes, along with COD reductions of 82% for RhB and 79% for MO.

Although Fe_2_O_3_/Bi_2_WO_6_-based photocatalysts have been documented in the literature for their applications in dye removal and antibiotics, we would like to highlight a few features of the current study that set it apart from earlier research and make incremental but significant contributions to the field. The nitroaromatic pollutant, *para*-nitrophenol (PNP), has received less attention in Fe_2_O_3_/Bi_2_WO_6_ photocatalysis studies. Initially, a low range of Fe_2_O_3_ loadings (1, 3, and 5 wt%) on hierarchical Bi_2_WO_6_ microspheres is systematically evaluated in this study for the degradation of PNP. Second, we offer a multi-technique structural and optical characterisation (XRD, Raman, UV-DRS, PL, and EDS mapping) that enables us to correlate photocatalytic performance with loading-dependent changes in band gap, charge-carrier recombination, and elemental distribution, providing a more detailed picture of how even low Fe_2_O_3_ loading affects the optoelectronic behavior of the composite. Third, and perhaps most significantly, this work goes beyond performance evaluation alone by combining the mechanistic study with green chemistry/green metrics analysis, placing the catalytic performance within a more comprehensive sustainability framework. Additionally, the effectiveness of this photocatalytic process was tested with groundwater samples.

## 2. Experimental section

### 2.1 Material synthesis

Analytical-grade bismuth(iii) nitrate pentahydrate (Bi(NO_3_)_3_·5H_2_O), sodium tungstate dihydrate (Na_2_WO_4_·2H_2_O) and iron(iii) nitrate nanohydrate (Fe(NO_3_)_3_·9H_2_O) were purchased from Sigma-Aldrich and used as such. The *p*-nitrophenol was purchased from Loba Chemie and utilised without additional purification. Distilled water was used in every experiment.

### 2.2 Synthesis of Bi_2_WO_6_ and Fe_2_O_3_/Bi_2_WO_6_ photocatalysts

A modified hydrothermal route, adapted from Xe *et al.*, 2019,^26^ was employed for the synthesis of the Fe_2_O_3_/Bi_2_WO_6_ hierarchical microsphere photocatalysts. Initially, 5 mmol of Bi(NO_3_)_3_·5H_2_O was dissolved in 15 mL of ethane-1,2-diol under continuous stirring to obtain solution A, while 2.5 mmol of Na_2_WO_4_·2H_2_O was dissolved in 15 mL of deionised water to form solution B, maintaining a molar Bi : W ratio of 2 : 1 consistent with the stoichiometry of Bi_2_WO_6_. These two solutions, A and B, were then combined under constant stirring at room temperature until a uniform white suspension was obtained. Subsequently, Fe(NO_3_)_3_·9H_2_O, corresponding to a Fe/Bi_2_WO_6_ molar ratio of 0.1, was dissolved in a small volume of water and added dropwise to the mixed suspension, followed by pH adjustment to 9.0 using dilute ammonia solution. The mixture was placed in a 100 mL Teflon-lined stainless-steel autoclave and hydrothermally treated at 180 °C for 5 h at autogenous pressure. The solid product was collected by centrifugation, washed several times with deionised water and ethanol, and dried at 80 °C in a vacuum oven for approximately 120 min after cooling to ambient temperature. Thereafter, the synthesized photocatalyst was heated in a muffle furnace for 4 h at 350 °C. To elucidate the influence of iron loading on the photocatalyst, two further composites with varying Fe/Bi_2_WO_6_ molar ratios of 0.3 and 0.5 were synthesised using the same protocol and designated as 3% Fe_2_O_3_/Bi_2_WO_6_ and 5% Fe_2_O_3_/Bi_2_WO_6_, respectively. On the other hand, pristine Bi_2_WO_6_ was also synthesized under identical conditions.

### 2.3 Catalyst characterization

Various comprehensive techniques were used to characterize the structural and spectral properties of the synthesized Bi_2_WO_6_ and Fe_2_O_3_/Bi_2_WO_6_ photocatalysts. To ascertain the phase purity and crystallinity, an X-ray diffraction (XRD) analysis was performed using Cu Kα radiation (*λ* = 1.5406 Å) at 45 kV and 40 mA on a PW3050/60 PANalytical X'Pert-PRO diffractometer. The surface morphology was investigated using field emission gun scanning electron microscopy (FEG-SEM), and the elemental composition was determined using energy-dispersive X-ray spectroscopy (Carl Zeiss Sigma 500 FEG-SEM with EDS). The SEM was operated at accelerating voltages between 0.5 and 35 kV. The optical properties were identified using UV-Vis diffuse reflectance spectroscopy (UV-2600, Shimadzu, Asia Pacific). High-resolution transmission electron microscopy (HRTEM) and selected-area electron diffraction (SAED) were performed using a Thermo Scientific Talos F200X G2 microscope at 200 kV. Interplanar *d*-spacings were directly determined from HRTEM lattice fringe pictures, and phase identification was validated by indexing the SAED diffraction rings. Raman spectroscopy (Make: Horiba, France, Model: Labram HR Confocal Micro-Raman Spectrometer) was used to identify the different crystalline phases and their relative abundance. Photoluminescence (PL) spectroscopy (Make: Horiba, France; Model: Labram HR Confocal Micro-Raman Spectrometer) was used to investigate electron–hole recombination in the synthesized photocatalysts. The specific surface area, pore volume, and pore size distribution of the synthesized photocatalysts were assessed using a Brunauer–Emmett–Teller (BET) surface area analyzer (Make: Smart Sorb 92/93, Smart Instruments Co). The electronic states of the synthesized samples were examined using a Thermo Fisher Scientific X-ray photoelectron spectrometer (XPS) equipped with a K ALPHA X-ray source. The PNP was quantified using a UV-Vis spectrophotometer at *λ*_max_ = 317 nm. To assess the mineralization of *p*-nitrophenol (PNP) under the ideal photocatalytic treatment conditions, total organic carbon (TOC) analysis was carried out using a TOC analyzer (Make: Elementar; Model: Enviro TOC). The stability and reusability of the photocatalyst were assessed through successive degradation cycles, and post-reaction X-ray diffraction (XRD) analysis was conducted after the recycling experiments to examine any changes in the crystalline structure of the recovered catalyst. Additionally, iron leaching was assessed using inductively coupled plasma (ICP-OES) analysis to assess catalyst stability.

### 2.4 Photocatalytic activity

Photocatalytic experiments were conducted in a laboratory-scale batch photoreactor (Fig. S1), using 200 mL of a 20 mg L^−1^ PNP solution irradiated with four Philips cool-white LEDs (20 W each, 400–700 nm), mounted in a custom-fabricated chamber. The incident light intensity at the solution surface was 9562 lux, measured using a calibrated lux meter. The reaction suspension was continuously stirred throughout the experiment using a magnetic stirrer (Remi 2MHI) to ensure uniform catalyst dispersion and homogeneous light exposure.

Prior to irradiation, the catalyst-PNP suspension was stirred in the dark for 30 min to attain adsorption–desorption equilibrium. The light source was then activated to initiate the photocatalytic reaction. Preliminary studies were done initially for 300 min. After 140 min, insignificant photocatalytic removal of PNP was observed; therefore, 140 min was chosen as the irradiation time for the latter part of the studies. At defined time intervals, 3 mL aliquots were withdrawn, filtered through PTFE membrane filters (0.22 µm, Millipore), and analyzed by UV-Vis spectrophotometry for the quantification of PNP removal. The effect of key operational parameters, including catalyst dosage, initial pH, and initial PNP concentration, was systematically investigated. All experiments were performed in triplicate, and the results are reported as mean values with acceptable reproducibility. PNP degradation efficiency (%) was calculated as shown in eqn (1) as follows:1
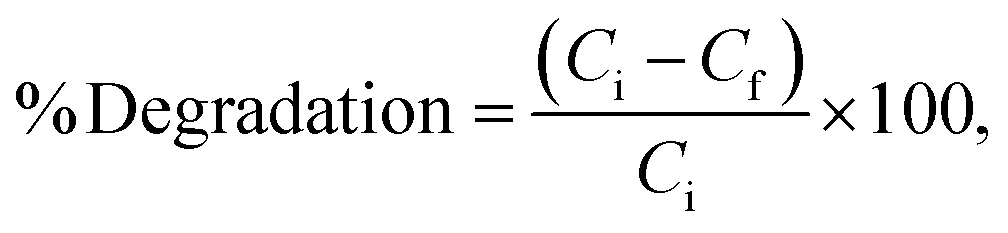
where *C*_i_ is the initial concentration and *C*_f_ is the concentration of PNP during the photocatalytic treatment.

### 2.5 Green chemistry metrics

The sustainability of the photocatalytic process was evaluated using five green chemistry metrics: mass intensity (MI), solvent/water intensity (SI), reaction mass efficiency (RME), environmental factor (E-factor), and specific energy consumption (M), in accordance with the framework established by Tripathy, H. *et al.*^27^ All metrics were assessed for the optimised degradation experiment (20 mg L^−1^ PNP, 250 mg L^−1^ catalyst, pH 7, 140 min of visible-light irradiation). MI was defined as the entire non-aqueous input mass (catalyst + PNP) divided by the mass of degraded PNP. SI was determined as the mass of deionised water utilised per unit mass of degraded PNP. RME was quantified as the proportion of non-aqueous input mass transformed into degraded output. The E-factor was calculated as the total non-aqueous waste per unit mass of degraded PNP. The specific energy consumption M (kWh kg^−1^) was estimated by dividing the total electrical energy input, derived from the nominal power rating of four 20 W visible-light LED lamps and the irradiation period (140 min), by the mass of PNP degraded in kg.

## 3. Results and discussion

### 3.1 Characterization studies

**Fig. 1:**
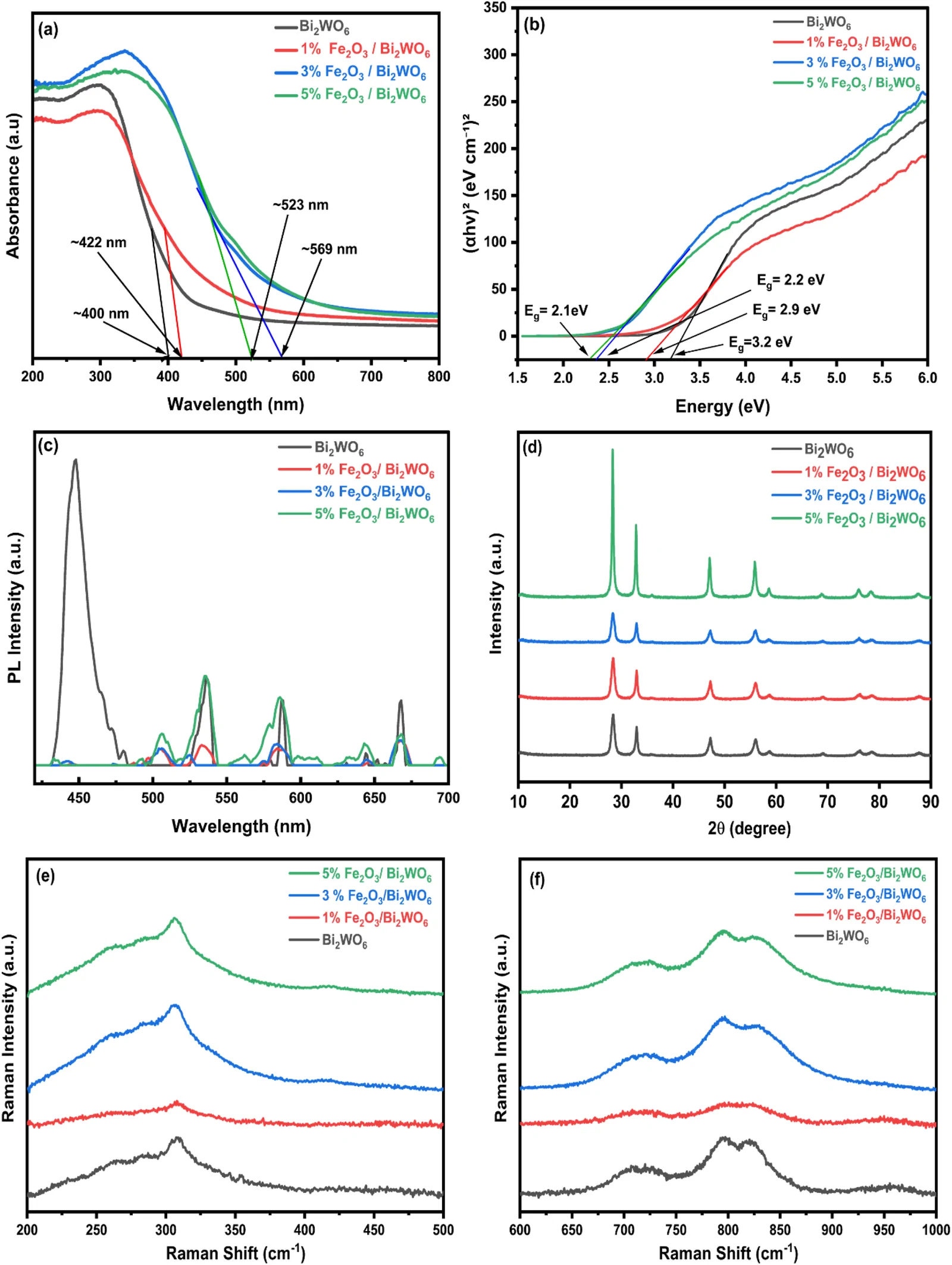
(a) UV-vis absorption spectra of pure Bi_2_WO_6_ and the Fe_2_O_3_/Bi_2_WO_6_ composites. (b) Estimated band gap energies of Bi_2_WO_6_ and Fe_2_O_3_/Bi_2_WO_6_. (c) Photoluminescence spectra of Bi_2_WO_6_ and Fe_2_O_3_/Bi_2_WO_6_ (1, 3, and 5 wt%). (d) XRD patterns of Bi_2_WO_6_ and Fe_2_O_3_/Bi_2_WO_6_ (1, 3, and 5 wt%). Raman spectra of Bi_2_WO_6_ and Fe_2_O_3_/Bi_2_WO_6_ (1, 3, and 5 wt%) in the ranges of (e) 200–500 cm^−1^ and (f) 500–1000 cm^−1^.

The optical properties of Bi_2_WO_6_ and the Fe_2_O_3_/Bi_2_WO_6_ composites were examined by UV-vis diffuse reflectance spectroscopy (200–800 nm, BaSO_4_ reference) as shown in Fig. 1a and b. Pristine Bi_2_WO_6_ displayed an absorption edge at ∼460 nm. Incorporation of Fe_2_O_3_ at 1, 3, and 5 wt% induced a systematic red shift, extending the absorption onset to ∼520 nm, attributed to Fe 3d mid-gap states, and narrowing the effective band gap, confirming the enhanced visible-light harvesting in all the composite samples. The optical band gaps of pure Bi_2_WO_6_, 1% Fe_2_O_3_/Bi_2_WO_6_, 3% Fe_2_O_3_/Bi_2_WO_6_, and 5% Fe_2_O_3_/Bi_2_WO_6_ were found to be 3.2 eV, 2.9 eV, 2.2 eV, and 2.1 eV, respectively.

The band gap of pure Bi_2_WO_6_ (3.2 eV) is slightly higher than the value of ∼2.8 eV reported by Fu, H. *et al.*,^28^ which may be attributed to differences in particle size and synthesis conditions. The progressive decrease in band gap upon Fe_2_O_3_ incorporation confirms the successful introduction of Fe^3+^ dopant states within the Bi_2_WO_6_ lattice, extending its optical absorption towards the visible region.^29^ This difference reflects the sensitivity of the band gap in d^0^ perovskites to the electronegativity of the transition metal ion, the connectivity of the metal–oxygen polyhedra, and the deviation of the M–O–M bond angle from linearity.^30^

Among 1%, 3% and 5% Fe_2_O_3_/Bi_2_WO_6_ photocatalysts, the 5% Fe_2_O_3_/Bi_2_WO_6_ photocatalyst exhibits the smallest band gap (2.1 eV) and thus the strongest visible-light absorption. However, a reduced band gap alone does not determine higher photocatalytic activity. Increased Fe concentrations result in PL spectra revealing a greater abundance of Fe-related defect states acting as recombination centers; simultaneously, XRD and Raman analyses indicate slight lattice distortion and possible Fe-rich clustering. Taken together, these phenomena promote electron–hole recombination and reduce the availability of effective WO_6_ surface sites, resulting in reduced PNP removal at higher Fe loading.^31^

Additionally, the specific physical form of the solid sample can markedly influence its optical properties.^16^ The photoluminescence (PL) spectra of all composites were obtained to examine the recombination dynamics of the photogenerated charge carriers (Fig. 1c). Pure Bi_2_WO_6_ demonstrated the most significant photoluminescence intensity at around 448 nm, indicating fast electron–hole recombination and poor charge separation efficiency. The weaker emission bands at ∼535 nm, ∼580 nm and ∼650 nm are assigned to the transitions of the defect states due to the oxygen vacancies, Bi–O/W–O lattice distortions and surface trap states of Bi_2_ WO_6_. The inclusion of Fe_2_O_3_ decreased all the emission bands considerably, showing that the p–n Fe_2_O_3_/Bi_2_WO_6_ Fe-doped Bi_2_WO_6_ hierarchical microspheres favor the quick interfacial transfer of photogenerated electrons from the CB of Bi_2_WO_6_ to Fe_2_O_3_, which spatially separates the charge carriers and suppresses the radiative recombination. Among all the composites, the 1% Fe_2_O_3_/Bi_2_WO_6_ composite exhibited the lowest photoluminescence intensity at the band edge emission (∼448 nm) and the defect-associated bands (535–665 nm), which is attributed to the best charge separation at this optimum concentration of Fe_2_O_3_. As the Fe_2_O_3_ content increased (3% and 5%), the intensity of photoluminescence gradually increased, and the defect-state emission bands at 535–665 nm became more intense, which can be attributed to the agglomeration of excess Fe_2_O_3_ particles that provide additional recombination centers and reduce the charge-separation efficiency. The results are in good accord with the photocatalytic degradation data, showing that 1% Fe_2_O_3_/Bi_2_WO_6_ had the highest photocatalytic activity among all the synthesized photocatalysts.^32^ X-ray diffraction patterns (Fig. 1d) of all the samples exhibited well-defined peaks at 2*θ* ≈ 28.5°, 32.8°, 47.1°, 55.7°, 58.5°, and 75.8°, indexed to the (131), (200), (202), (133), (262), and (391) planes of orthorhombic Bi_2_WO_6_ (ICDD PDF# 01-076-2478), confirming the formation of a phase-pure *Aurivillius*-type structure with good crystallinity. No discrete diffraction peaks attributable to α-Fe_2_O_3_ (ICDD PDF# 00-338-1285) were detected in any Fe_2_O_3_/Bi_2_WO_6_ composite, indicating that iron species are either uniformly dispersed as nanosized particles below the XRD detection threshold or exist in close structural association with the Bi_2_WO_6_ lattice.^33^ The Fe-doped composites displayed marginally enhanced Bi_2_WO_6_ reflection intensities relative to pristine Bi_2_WO_6_, suggesting improved crystallinity during hydrothermal synthesis. A subtle shift of the (131) reflection toward lower 2θ values in Fe-doped samples indicates minor lattice expansion, consistent with partial substitution of Bi^3+^ sites by Fe^3+^ ions or surface-associated lattice distortion. Such *d*-spacing augmentation following Fe incorporation has been documented in the literature^34^ and is typically attributed to robust interfacial contact between the metal oxide dopant and the Bi_2_WO_6_ matrix. Collectively, the enhanced crystallinity, which reduces bulk defects serving as recombination centres and extends charge-carrier lifetimes, and the Fe-induced lattice distortion, which generates electronic states and interfacial charge-transfer sites acting as electron traps, are anticipated to synergistically promote photocatalytic activity.^35^ Raman analysis (Fig. 1e and f) was performed to further characterise the phase structure of the synthesised photocatalyst. The distinctive vibrational bands of orthorhombic Bi_2_WO_6_ can be seen clearly in three main areas: (i) 200–500 cm^−1^, which are due to the internal bending vibrations of WO_6_ octahedra and Bi–O bending modes; and (ii) 600–1000 cm^−1^, which are due to the stretching vibrations of W–O bonds in both terminal and bridging tungstate groups.^36^ The peaks at about 95 cm^−1^ and 305 cm^−1^ are associated with the translational and bending modes of the Bi^3+^ and WO_6_ units. The band at around 710 cm^−1^ is caused by the antisymmetric stretching vibration of bridging W–O bonds.^37^ The two peaks at about 790 and 820 cm^−1^ are caused by the antisymmetric and symmetric stretching modes of terminal O–W–O groups, respectively.^38^ The Fe_2_O_3_/Bi_2_WO_6_ composites (1, 3, and 5 wt%) showed small but noticeable changes compared to pure Bi_2_WO_6_. As the concentration of Fe increased, the distinctive bands at about 95 cm^−1^, 305 cm^−1^, and 790 cm^−1^ shifted to the blue end of the spectrum. This shows that the lattice was distorted and that the W–O bond interactions were stronger after Fe was added. Also, the peaks were bigger, especially in the high-frequency stretching region (600–1000 cm^−1^), when the Fe doping went from 1% to 5%. This suggests greater local disorder and that defects (such as oxygen vacancies) could have formed within the lattice, which is consistent with the results of the XRD study.^39^ The changes in the Raman spectrum show that Fe ions are added to the Bi_2_WO_6_ lattice instead of forming separate oxide phases. The changes in the observed bands and their broadening with increasing Fe content indicate that doping alters the coordination of Bi–O and W–O bonds in the vicinity. These changes to the structure are in line with those in the electrical structure caused by defects, which could improve the photocatalytic activity.^40^

**Fig. 2:**
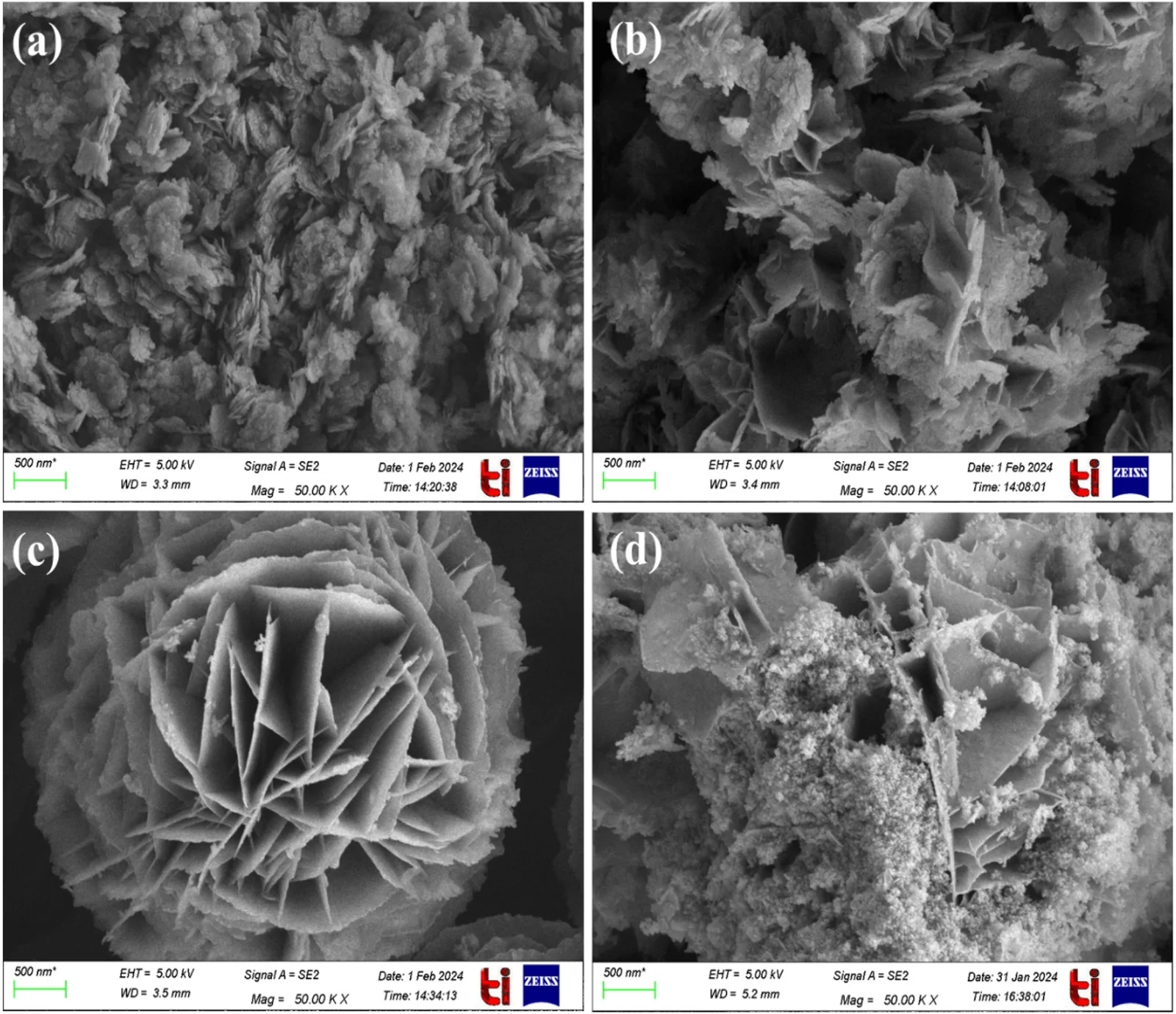
SEM images of (a) Bi_2_WO_6_, (b) 1% Fe_2_O_3_/Bi_2_WO_6_, (c) 3% Fe_2_O_3_/Bi_2_WO_6_, and (d) 5% Fe_2_O_3_/Bi_2_WO_6_.

SEM and EDS analysis (Fig. 2 and S3) were used to determine the morphology and composition of the Bi_2_WO_6_ and Fe_2_O_3_/Bi_2_WO_6_ photocatalyst. The SEM image of Bi_2_WO_6_ revealed a highly porous, layered morphology, suggesting a flake-like or nanosheet structure. The microsphere is constructed from smooth nanoplates *via* self-assembly and Ostwald ripening.^41^ As a result, Bi_2_WO_6_ features numerous pores among its overlapping nanosheets, facilitating the transmission of small molecules. This porous architecture promotes the movement of reactant and product molecules into and out of the material, thereby enabling chemical reactions to proceed more efficiently.^42^ With the addition of 1% iron to the Bi_2_WO_6_, the flake-like structure started to agglomerate. This agglomerated structure started to take the shape of a ball.^43^ With an increase in iron to 3%, it developed a well-defined, spherical morphology with intricate, layered structures, giving it a microflower-like appearance. The composite maintained its hierarchical flower-like superstructure, as low-temperature calcination did not induce any significant morphological changes. Sparse protruding islands were observed on the nanosheet surfaces, likely corresponding to Fe_2_O_3_ nanoparticles formed during impregnation of Bi_2_WO_6_ with Fe(NO_3_)_3_·9H_2_O followed by thermal treatment.^44^ The spheres appear to be composed of thin, sheet-like structures arranged in a hierarchical pattern. The uniformity in size and structure indicates controlled growth, likely influenced by synthesis conditions. As the iron content was increased to 5% in Fe_2_O_3_/Bi_2_WO_6_, the spherical structure appeared more compact, suggesting that agglomeration affects charge separation and recombination dynamics (earlier discussed in the PL analysis).^45^

EDS spectra (Fig. S3 in the SI) reveal the presence of all the components used to synthesize the Fe_2_O_3_/Bi_2_WO_6_. All composites were found to include Bi, W, O, and Fe, with Fe content increasing from 1% to 5% and uniform Bi : W : O ratios indicating consistent elemental distribution without phase segregation, according to EDS. Also, mapping confirms that the Fe signal is spatially co-located with O, W, and Bi over the whole sample surface at all loadings, including 1 wt%, showing that Fe is chemically present and homogeneously diffused on the Bi_2_WO_6_ matrix rather than being separated into huge isolated clusters.

**Fig. 3:**
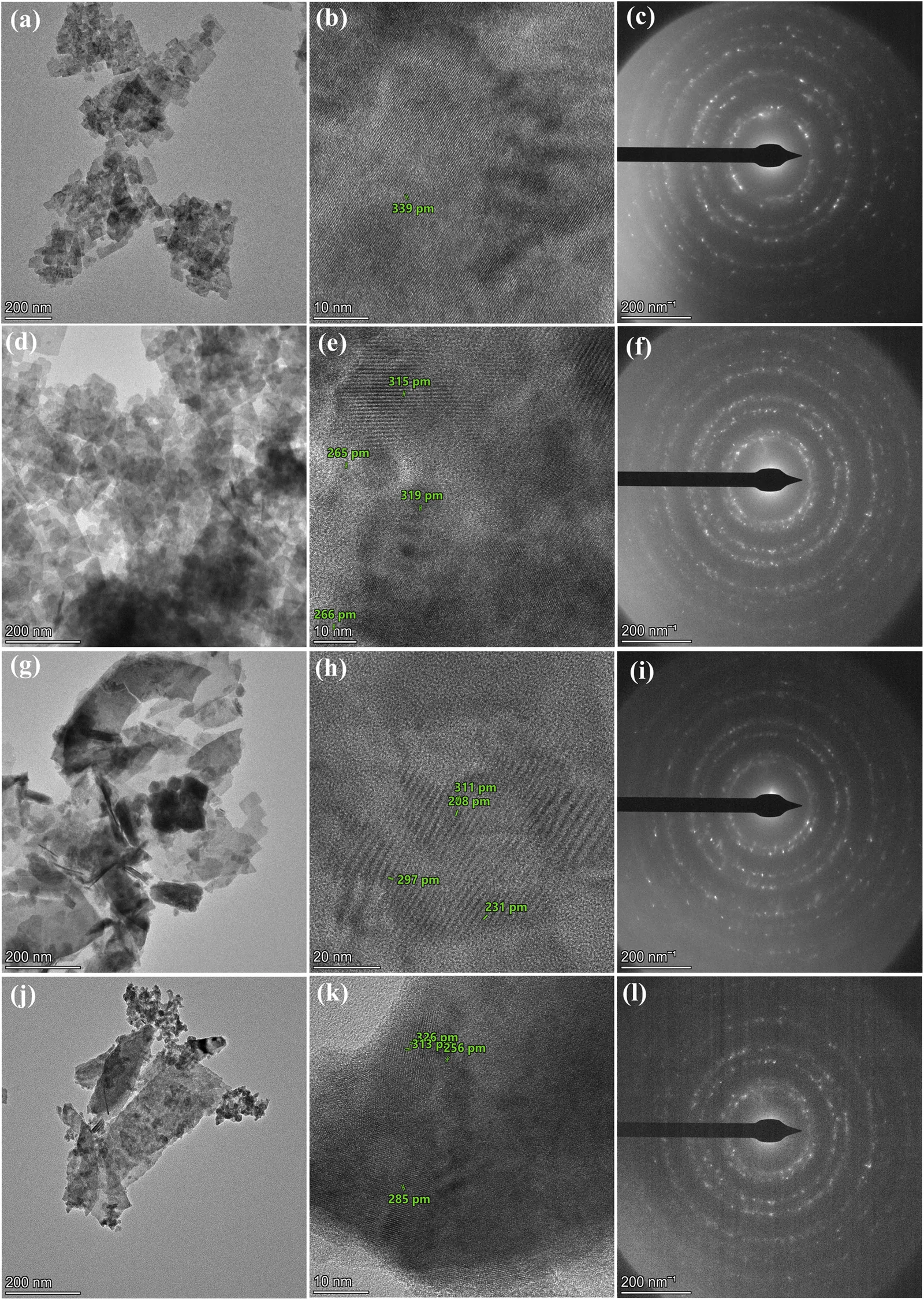
HRTEM images and SAED patterns of (a–c) Bi_2_WO_6_ and the (d–f) 1% Fe_2_O_3_/Bi_2_WO_6_, (g–i) 3% Fe_2_O_3_/Bi_2_WO_6_, and (j–l) 5% Fe_2_O_3_/Bi_2_WO_6_ composites under different magnifications.

The microstructural characteristics, local crystallinity, and phase composition of pristine Bi_2_WO_6_ and the Fe_2_O_3_/Bi_2_WO_6_ composite photocatalysts were analyzed using high-resolution transmission electron microscopy (HRTEM) with selected-area electron diffraction (SAED) (Fig. 3). Collectively, these methodologies furnish atomic-scale evidence for the *Aurivillius*-type crystal structure of the Bi_2_WO_6_ host, the characteristics and distribution of the Fe_2_O_3_ phase, and the establishment of a close Fe-doped Bi_2_WO_6_ hierarchical microspheres interface, all of which are essential factors influencing photocatalytic charge-carrier dynamics.^46^ HRTEM images of pristine Bi_2_WO_6_Fig. 3a exhibit a nanosheet shape with lateral dimensions ranging from roughly 80 to 200 nm, indicative of the *Aurivillius* layered perovskite structure of this material. The nanosheets have a propensity for self-assembly into loosely aggregated, flower-like hierarchical structures, influenced by robust interlayer contacts stemming from the alternating (Bi_2_O_2_)^2+^ fluorite and (WO_4_)^2−^ perovskite layers within the Bi_2_WO_6_ crystal structure.^47^

Fig. 3b, showing pristine Bi_2_WO_6_, clearly reveals uninterrupted lattice fringes spanning the nanosheet domains, thereby affirming the substantial crystalline order of the hydrothermally produced material. Two separate sets of lattice fringes are identified: a main set with an interplanar spacing of *d* = 0.339 nm, corresponding to the (131) crystallographic plane of orthorhombic Bi_2_WO_6_ (ICDD PDF# 01-076-2478), and a secondary set with *d* ≈ 0.258 nm, attributed to the (200) plane of Bi_2_WO_6_. The observed *d*-values align exceptionally well with the *d*-spacings derived from Bragg's law for the respective XRD reflections at 2*θ* = 28.5° and 32.8°, thereby offering independent atomic-scale validation of the phase-pure orthorhombic structure.^48^

The SAED pattern of pure Bi_2_WO_6_ (Fig. 3c) features concentric diffraction rings with bright, distinct spots throughout the ring perimeter, indicative of polycrystalline material with sizable, well-ordered crystalline domains displaying partial preferred orientation.^49^ Four primary rings are identified, indexed from the innermost to the outermost as the (131), (200), (202), and (133) planes of orthorhombic Bi_2_WO_6_, with corresponding *d*-spacings of roughly 0.312, 0.273, 0.195, and 0.160 nm, respectively. The lack of a diffuse amorphous halo or unassigned rings in the SAED pattern verifies the phase purity and high crystallinity of the pure Bi_2_WO_6_, as evidenced by the sharp, well-resolved XRD reflections.^50^ The HRTEM image (Fig. 3d) of the 1% Fe_2_O_3_/Bi_2_WO_6_ composite confirms that the nanosheet architecture of the Bi_2_WO_6_ host is maintained after the addition of Fe at a 1 wt% loading, with no discernible structural collapse or significant morphological change in the host framework. It also demonstrates (Fig. 3e) distinctly resolved Bi_2_WO_6_ lattice fringes with *a* spacing of about 0.339 nm, aligning with the (131) plane of orthorhombic Bi_2_WO_6_ and extending uniformly across the nanosheet areas. No distinct lattice fringes corresponding to a separate crystalline α-Fe_2_O_3_ phase are observable in the HRTEM images of the 1% composite, suggesting that at this minimal loading level, Fe species are present as highly dispersed, sub-nanometric or partially amorphous entities at or near the surface of the Bi_2_WO_6_ nanosheets, beneath the spatial resolution limit for lattice fringe detection by HRTEM. The SAED pattern of the 1% Fe_2_O_3_/Bi_2_WO_6_ composite (Fig. 3f) closely mirrors that of pure Bi_2_WO_6_, exhibiting four concentric patchy rings corresponding to the Bi_2_WO_6_ (131), (200), (202), and (133) planes (*d* = 0.312, 0.273, 0.195, and 0.160 nm). No further diffraction rings corresponding to α-Fe_2_O_3_ are observed, which aligns perfectly with the XRD (Fig. 1d) findings, indicating the absence of hematite Bragg reflections in the composite patterns at this composition. The ring pattern of the 1% composite exhibits marginally sharper and slightly more intense features compared to pristine Bi_2_WO_6_, indicating a modest enhancement in Bi_2_WO_6_ crystallinity due to low-level Fe incorporation during hydrothermal synthesis, as supported by the increased XRD reflection intensities.^51^

HRTEM images of the 3% Fe_2_O_3_/Bi_2_WO_6_ composite (Fig. 3g) demonstrate the preservation of the Bi_2_WO_6_ nanosheet shape, accompanied by a significantly increased surface density of nanoscale features relative to the 1% sample, indicative of a more substantial deposition of Fe_2_O_3_ on the Bi_2_WO_6_ surface at this concentration. The HRTEM analysis of the 3% composite demonstrates two distinct sets of lattice fringes within the same field of view: a predominant set with *d* ≈ 0.339 nm, corresponding to the (131) plane of Bi_2_WO_6_, and a secondary set with *d* ≈ 0.258 nm, attributable to the (110) plane of α-Fe_2_O_3_ (ICDD PDF# 00-33-0664, *d* = 0.252 nm). The simultaneous observation of Bi_2_WO_6_ and α-Fe_2_O_3_ lattice fringes in close spatial proximity at the interfacial region between the two phases offers direct atomic-scale evidence for the formation of Fe_2_O_3_/Bi_2_WO_6_ Fe-doped Bi_2_WO_6_ hierarchical microspheres, a structural characteristic essential for effective interfacial photogenerated charge transfer. A nuanced expansion of the Bi_2_WO_6_ fringe contrast at the interface, compared to the unaltered sample, indicates local lattice strain due to the structural incongruity at the Fe-doped Bi_2_WO_6_ hierarchical microspheres boundary, corroborated by the minor shift of the (131) XRD reflection toward reduced 2θ values in the Fe-doped composites.^52^

The HRTEM image and SAED pattern of the 3% Fe_2_O_3_/Bi_2_WO_6_ composite (Fig. 3g–i) exhibit a more intricate ring structure compared to both pure Bi_2_WO_6_ and the 1% composite, characterized by a greater density of distinct diffraction spots and an elevated number of discernible rings. The four primary Bi_2_WO_6_ rings (131), (200), (202), and (133) are preserved with significant intensity, affirming the integrity of the orthorhombic host structure. Moreover, additional rings corresponding to the (104) (*d* = 0.270 nm) and (110) (*d* ≈ 0.252 nm) planes of α-Fe_2_O_3_ are observable at reciprocal radii marginally exceeding the Bi_2_WO_6_ (200) ring, indicating the emergence of identifiable α-Fe_2_O_3_ crystalline domains in the SAED analysis. The appearance of Fe_2_O_3_ diffraction features at 3% loading, which are not present at 1%, signifies a compositional barrier beyond which Fe_2_O_3_ nanodomains of enough size and crystallinity for electron diffraction detection are formed on the Bi_2_WO_6_ surface.^53^

TEM pictures of the 5% Fe_2_O_3_/Bi_2_WO_6_ composite (Fig. 3j and k) demonstrate the preservation of the Bi_2_WO_6_ nanosheet architecture, exhibiting a significantly increased surface coverage of Fe_2_O_3_-related contrast features compared to the lower-loading composites. The HRTEM imaging of the 5% composite distinctly shows both Bi_2_WO_6_ and α-Fe_2_O_3_ lattice fringes, with *d* ≈ 0.339 nm for Bi_2_WO_6_ (131) and *d* ≈ 0.258 nm for α-Fe_2_O_3_ (110), exhibiting greater frequency and enhanced fringe contrast compared to the 3% sample, indicative of the increased volume fraction and average domain size of the Fe_2_O_3_ phase at higher loading levels. A significant rise in local fringe irregularity and lattice distortion within the Bi_2_WO_6_ domains near the Fe_2_O_3_ nanodomains is noted, attributed to the increased lattice strain caused by the higher density of Fe_2_O_3_/Bi_2_WO_6_ interfacial contacts at 5 wt% loading; this atomic-scale distortion correlates directly with the notable shift of the (131) XRD reflection and the slight broadening of diffraction peaks at this composition.^54^ The SAED pattern of the 5% Fe_2_O_3_/Bi_2_WO_6_ composite (Fig. 3l) is the most intricate in the series, exhibiting a dense configuration of diffraction spots arranged over many concentric rings from both phases. The Bi_2_WO_6_ (131), (200), (202), and (133) rings are distinctly observable, while the α-Fe_2_O_3_ rings associated with the (104), (110), and (113) planes (*d* ≈ 0.270, 0.252, and 0.221 nm, respectively) are clearly delineated and exhibit higher relative intensity compared to the 3% sample, aligning with the increased quantity and crystallinity of Fe_2_O_3_ at this concentration. No extra unassigned rings are observed in the SAED pattern of the 5% composite. The irregular appearance of all the rings in the series indicates the well-crystallized, textured domain structure of the Bi_2_WO_6_ nanosheets preserved throughout the composite series, which facilitates anisotropic charge transfer and enhances photocatalytic performance.^24^ The HRTEM lattice fringe spacings and SAED ring indexing results for all the samples align perfectly with the XRD study, offering complementary multi-scale structural confirmation. The principal Bi_2_WO_6_ lattice spacing of *d* ≈ 0.339 nm, derived from HRTEM fringes, accurately corresponds to the *d*-value computed from Bragg's law for the (131) XRD reflection at 2*θ* ≈ 28.5°. Additionally, the *d* ≈ 0.258 nm spacing is consistent with the (200) reflection at 2*θ* ≈ 32.8° (*d*_calc_ = 0.273 nm, within permissible experimental measurement error). The SAED ring radii for the four Bi_2_WO_6_ rings, indexed as (131), (200), (202), and (133), correspond to the *d*-spacings calculated from the identical XRD reflections at 2*θ* = 28.5°, 32.8°, 47.1°, and 55.7°, respectively, thereby offering independent reciprocal-space validation of the phase designation. The lack of α-Fe_2_O_3_ diffraction characteristics in both XRD and SAED at 1% loading, alongside their simultaneous appearance at 3–5% loading in both methods, creates a coherent narrative: Fe_2_O_3_ is present as dispersed, non-crystalline entities at low concentrations and gradually nucleates into observable crystalline nanodomains beyond a certain threshold of Fe loading. The minor displacement of the XRD (131) reflection to lower 2*θ*, indicative of Bi_2_WO_6_ lattice expansion, is substantiated at the atomic level by the broadening of (131) HRTEM fringe contrast and heightened local lattice disorder in composites with elevated Fe content, thereby establishing a coherent narrative of lattice distortion across both diffraction and imaging methodologies.^55^ The XRD, HRTEM, and SAED data collectively present a coherent multi-scale structural representation of phase-pure orthorhombic Bi_2_WO_6_ nanosheets containing closely interfaced α-Fe_2_O_3_ nanodomains, exhibiting progressive Fe-doped Bi_2_WO_6_ hierarchical microsphere formation, lattice distortion, and enhanced crystallinity as Fe loading increases from 1 to 5 wt%.^56^

To further investigate the surface chemical state of the 1% Fe_2_O_3_/Bi_2_WO_6_, high-resolution XPS spectra (Fig. S4) of the Bi 4f, W 4f and O 1s core levels were employed. The Bi 4f_7/2_ and Bi 4f_5/2_ spin–orbit components are found at ∼158.9 and ∼164.3 eV, respectively, which is essentially unshifted from the values reported for pristine Bi_2_WO_6_, indicating that the original Bi–O coordination environment is mostly preserved, and the Fe incorporation does not perturb the Bi_2_WO_6_ sublattice to a significant extent. These results, in conjunction with the elemental mapping, suggest a heterojunction configuration where Fe_2_O_3_ is finely and homogeneously distributed on the surface of Bi_2_WO_6_, rather than embedded inside the bulk lattice. The elemental maps (Fig. S3) also indicate a uniform spatial overlap among Fe, Bi, W, and O. This close, continuous interfacial contact between the two semiconductor phases is an essential structural feature for efficient interfacial charge transfer and the suppression of electron–hole recombination. Taken together, the XPS and EDS elemental mapping data corroborate the successful surface-level integration of Fe_2_O_3_ within the Bi_2_WO_6_ matrix.

BET analysis elucidates the surface area and pore volume of the synthesized catalysts, wherein an increased surface area offers additional active sites, hence augmenting photocatalytic activity. All the photocatalysts exhibit Type IV(a) isotherms with a H1 hysteresis loop (IUPAC, 2015), indicating a distinct mesoporous architecture characterized by homogeneous cylindrical pores and minimal network effects. These features for all the Fe_2_O_3_/Bi_2_WO_6_ composites are tabulated in Table 1.

**Table 1:** Surface area and pore volume of the Bi_2_WO_6_ and Fe_2_O_3_/Bi_2_WO_6_ composites obtained from their N_2_ adsorption–desorption isotherms

Photocatalyst	Isotherm type (IUPAC, 2015)	Hysteresis type	Specific surface area (m^2^ g^−1^)	Average pore diameter (nm)	Pore volume (cm^3^ g^−1^)
Bi_2_WO_6_	IV(a)	H1	29.9	13.783	0.1029
1% Fe_2_O_3_/Bi_2_WO_6_	IV(a)	H1	30.415	11.937	0.0908
3% Fe_2_O_3_/Bi_2_WO_6_	IV(a)	H1	7.79	7.843	0.0196
5% Fe_2_O_3_/Bi_2_WO_6_	IV(a)	H1	12.872	12.975	0.0412

Pristine Bi_2_WO_6_ possesses a specific surface area of 29.9 m^2^ g^−1^, an average pore diameter of 13.78 nm, and a pore volume of 0.1029 cm^3^ g^−1^, demonstrating a well-developed mesoporous structure conducive to photocatalysis. The 1% Fe_2_O_3_/Bi_2_WO_6_ composite maintains a similar surface area (29.4 m^2^ g^−1^) and pore volume (0.0908 cm^3^ g^−1^), with a slightly diminished pore diameter (11.94 nm), reaching an ideal equilibrium between surface accessibility and pore architecture for improved adsorption and catalytic efficacy.^57^ At 3% Fe_2_O_3_ loading, a distinct decrease in specific surface area (7.79 m^2^ g^−1^), pore width (7.84 nm), and pore volume (0.0196 cm^3^ g^−1^) indicates pore constriction or partial obstruction due to excess Fe_2_O_3_. A limited recovery at 5% loading (specific surface area = 12.87 m^2^ g^−1^; pore diameter = 12.98 nm; pore volume = 0.0412 cm^3^ g^−1^) presumably indicates pore merging rather than genuine mesostructure regeneration.^58^ The results indicate that the Fe_2_O_3_ concentration significantly influences the pore structure and surface area of Bi_2_WO_6_ composites, with 1% loading being optimal for enhancing active site density and mass transport efficiency.^44^

### 3.2 Effect of different operating parameters on PNP degradation

The photocatalytic experiments were performed using Bi_2_WO_6_ and Fe_2_O_3_/Bi_2_WO_6_ (1, 3 and 5%) synthesised for the degradation of PNP. Various parameters, such as pH, catalyst dose, and PNP concentration, were selected to conduct the photocatalytic experiments and determine the degradation of PNP. The rate-constant (*k*) plots obtained under varying operational parameters are presented in the SSI (Fig. S5).

A 30-minutes dark equilibration phase was conducted before visible-light exposure to distinguish surface-adsorption influences from the photocatalysis. Under optimized conditions (250 mg L^−1^ catalyst, pH 7, 20 mg L^−1^ PNP), 84.5% removal of PNP was achieved in 135 min. Of this total 84.5% PNP removal, the adsorption in the dark contributed 37.6% removal, and ∼46.9% removal (after adsorption in the dark) occurred through visible-light-driven photocatalysis. This aligns with the increased surface area associated with the nanosheet structure of the synthesized photocatalyst and is further corroborated by SEM analysis (Fig. 2).

#### 3.2.1. Catalyst suitability

During the preliminary experiments with Bi_2_WO_6_ and Fe_2_O_3_/Bi_2_WO_6_ (1%, 3%, and 5%), at pH 7.45 (natural pH of the solution), 20 ppm PNP, and 250 mg L^−1^ catalyst dosage, 1% Fe_2_O_3_/Bi_2_WO_6_ demonstrated the best photocatalytic efficiency for PNP degradation among the synthesized photocatalysts (see Fig. S2 in the SI). Therefore, further experiments for the effect of operating parameters on the removal of PNP were conducted with 1% Fe_2_O_3_/Bi_2_WO_6_. Improved charge separation at low Fe_2_O_3_ loading might be the cause of this increased activity. On the other hand, excessive Fe_2_O_3_ may promote particle clumping and block active sites, thereby reducing the photocatalytic efficiency. Fe-modified Bi_2_WO_6_ photocatalysts have been shown to exhibit similar behavior.^40^

The enhanced photocatalytic activity of the 1% Fe_2_O_3_/Bi_2_WO_6_ sample is supported by the interfacial interaction between Fe_2_O_3_ and Bi_2_WO_6_ as confirmed by the EDS elemental co-localization, band gap narrowing and PL quenching, which are all indicative of improved charge-carrier separation. Furthermore, the exceptional efficacy of the 1% Fe_2_O_3_/Bi_2_WO_6_ composite is attributed to a harmonious interplay between structural integrity and advantageous electrical alterations. The N_2_ adsorption–desorption data presented in Table 1 illustrate that pristine Bi_2_WO_6_ has a specific surface area of 29.9 m^2^ g^−1^ and a pore volume of 0.1029 cm^3^ g^−1^, whereas the 1% Fe_2_O_3_/Bi_2_WO_6_ maintains a similar surface area (30.4 m^2^ g^−1^) with a somewhat decreased pore volume (0.0908 cm^3^ g^−1^). Conversely, elevated Fe concentrations (3% and 5%) result in a significant deterioration and only a partial restoration of porosity (7.79 and 12.87 m^2^ g^−1^; 0.0196 and 0.0412 cm^3^ g^−1^), indicative of pore obstruction and agglomeration. Minimal Fe concentrations yield insufficient defect/trap sites to significantly enhance charge separation, whereas high Fe levels (≥2%) are recognized for creating recombination centers and obstructing active sites in Fe-Bi_2_WO_6_ systems. Thus, 1% Fe signifies an ideal loading level where Fe^3+^-induced oxygen vacancies and band-structure modulation improve visible-light absorption and charge-carrier separation while maintaining surface area accessibility, resulting in superior photocatalytic performance.^59^

#### 3.2.2. Initial pH

**Fig. 4:**
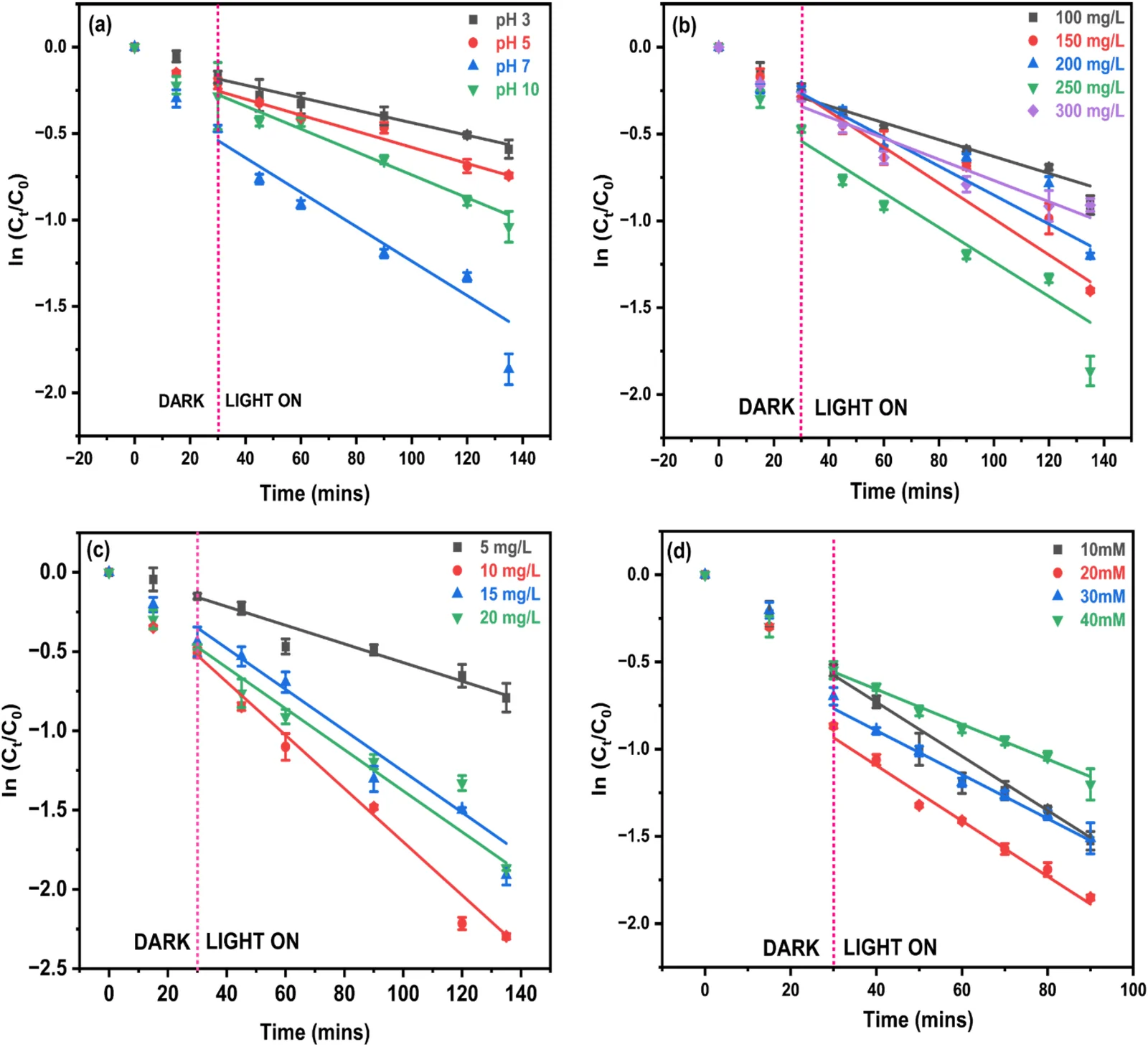
Pseudo-first-order kinetic curves of PNP degradation under different operating parameters: (a) initial pH values of 3, 5, 7, and 10, (b) 1% Fe_2_O_3_/Bi_2_WO_6_ dosages of 100–300 mg L^−1^, (c) initial PNP concentrations of 5, 10, 15, and 20 mg L^−1^, and (d) influence of H_2_O_2_ dosages of 10–40 mM.

The influence of initial solution pH (Fig. 4a) on the photocatalytic degradation of PNP was investigated over the range pH 3–10. The removal efficiencies of PNP were ordered as follows: pH 7 (84.5%) > pH 10 (64.7%) > pH 5 (52.4%) > pH 3 (44.6%), with associated pseudo-first-order rate constants of 0.01282, 0.0073, 0.00466, and 0.00385 min^−1^, respectively, demonstrating that neutral pH 7 provides optimal conditions for photocatalytic degradation. Consistently, the suspension at pH 7 attained the lowest ln (*C*_*t*_/*C*_0_) value after irradiation, whereas both acidic and strongly alkaline media exhibited smaller absolute slopes and higher residual PNP concentrations, evidencing slower intrinsic reaction rates. This pH dependence can be rationalised through three concurrent effects. First, the point of zero charge (pH_PZC_) of Bi_2_WO_6_-based composites typically lies in the range 5–6; at pH 3–5, the positive charge on the catalyst surface, together with proton-saturated surface hydroxyl groups, reduces the flux of ·OH radicals available for PNP oxidation.^25^ Second, PNP is a weakly acidic phenol (p*K*_a_ 7.15) that exists predominantly as the neutral molecule below pH 7; near-neutral pH aligns the catalyst surface charge with the most favourable adsorption orientation for PNP, maximising the interfacial pollutant concentration available for direct hole oxidation. Third, at pH 10, PNP is partially deprotonated to the *p*-nitrophenolate anion, and electrostatic repulsion between the anionic pollutant and the negatively charged catalyst surface suppresses adsorption at active sites, reducing the surface-bound pollutant concentration available for ·OH attack.^60^ Although pH 10 achieves a similar final conversion to pH 7 at 120 min, its significantly lower *k*_app_ confirms a slower intrinsic reaction rate under alkaline conditions. On the basis of these results, pH 7 was identified as the optimal reaction pH.

#### 3.2.3 Photocatalyst dose

The synthesized photocatalyst dosage was tested from 100 to 300 mg L^−1^ at pH 7 during 140 min of visible-light irradiation. Fig. 4b shows that photocatalytic efficiency exhibited an increasing trend from 59.8% (100 mg L^−1^, *k*_app_ = 0.0048 min^−1^) to 84.5% (250 mg L^−1^, *k*_app_ = 0.0093 min^−1^), thereafter removal efficiency declined at 300 mg L^−1^ (*k*_app_ =0.0060 min^−1^) catalyst dose.

The increase in removal of PNP due to the increase in catalyst dosage from 100 to 250 mg L^−1^ is due to the consequent increase in the number of photoactive surface sites for visible-light absorption and reactive oxygen species (·OH, ·O_2_^−^) production. Increased catalyst loading also increases the overall Fe_2_O_3_/Bi_2_WO_6_ Fe-doped Bi_2_WO_6_ hierarchical microsphere contact area within the irradiation volume, thereby improving the rate of interfacial charge separation and radical-mediated PNP oxidation. Exceeding the optimal concentration of 250 mg L^−1^ results in two opposing effects that hinder the performance: (i) excessive turbidity in the suspension decreases the penetration depth of visible light, thereby diminishing the photoactivated portion of the catalyst; and (ii) interparticle aggregation reduces the available specific surface area per unit mass, leading to a decrease in effective active-site density.^61^ Consequently, 250 mg L^−1^ was determined to be the best catalyst loading for the subsequent investigation.

#### 3.2.4. Concentration of PNP

The initial PNP concentration (Fig. 4c) was varied from 5 to 20 mg L^−1^ at the optimized catalyst loading of 250 mg L^−1^ and pH 7 for 140 min. In the experimentally validated concentration range of 5–20 mg L^−1^, a negative correlation between the initial PNP concentration and the pseudo-first-order rate constant was consistently noted, aligning with the Langmuir–Hinshelwood model for heterogeneous photocatalysis. This tendency is ascribed to the saturation of active sites on the catalyst surface at elevated pollutant concentrations, which constrains the production and interaction of reactive oxygen species (–OH, h^+^) with PNP molecules, thereby diminishing the overall degradation efficiency. At low substrate concentrations, the number of active surface sites surpasses the availability of the pollutant, facilitating fast and unobstructed ·OH-mediated oxidation.^62^

As the concentration rises, the gradual saturation of active sites by PNP molecules and oxidative intermediates diminishes the proportion of sites available for O_2_ reduction (·O_2_^−^ generation) and H_2_O oxidation (·OH generation).^63^ Simultaneously, the increased optical density of the solution reduces the penetration of incident visible light, thereby decreasing the photon flux at the catalyst surface.^64^ The nearly two-fold reduction in k_app_ from 20 mg L^−1^ (0.0130 min^−1^) highlights the system's sensitivity to substrate saturation. A working concentration of 20 mg L^−1^ was maintained as the standard condition, offering a realistic mid-range level of environmental pollutants suitable for precise kinetic measurement.^65^

#### 3.2.5. Influence of H_2_O_2_ dosage

To improve the degradation of PNP, H_2_O_2_ was added as an oxidizing agent. The impact of exogenous H_2_O_2_ (Fig. 4d) was assessed at concentrations of 10, 20, 30, and 40 mM under optimum conditions of pH 7, a catalyst dose of 250 mg L^−1^, and *C*_0_ = 20 mg L^−1^. With the addition of 20 mM H_2_O_2_, comparable removal efficiency (83.6%) was achieved within only 60 min, with increased reaction rate k_app = 0.0159 min^−1^. This demonstrates a substantial reduction (55.6%) in the treatment time required to degrade PNP in comparison with the photocatalyst alone.

Beyond this threshold, excess H_2_O_2_ acted as a hydroxyl radical (–OH) scavenger *via* the reaction (H_2_O_2_ + −OH → −OOH + H_2_O in which the produced hydroperoxyl radicals (–OOH) exhibit considerably lower oxidation potential than –OH, thus competitively reducing the primary reactive oxygen species and decreasing the overall degradation rate. At suboptimal values (10 mM), the limiting supply of ·OH from the exogenous oxidant reduced degradation to around 76%. The performance enhanced significantly at 20 mM, attributed to synergistic ·OH production from both direct photocatalytic water oxidation and heterogeneous Fenton-type reactions at the Fe_2_O_3_ surface (Fe^2+^/Fe^3+^ redox cycle).^66^ At 30 mM, the degradation profile remained comparable to that at 10 mM, with ∼74–75% removal after 60 minutes, suggesting that any additional ·OH generated at this dose is partially offset by emerging scavenging effects and competitive consumption of radicals. At 40 mM, the degradation diminished, aligning with traditional ·OH scavenging by surplus H_2_O_2_ (eqn (2)) as follows:

2 H_2_O_2_ + OH → −HO + H_2_O (*k* = 2.7× 10^7^ M^−1^ s^−1^),

where the generated hydroperoxyl radical (HO_2_·) is far less oxidising than ·OH. The significantly elevated dark-adsorption efficiencies at 30 and 40 mM further suggest surface-mediated dark Fenton activity at the Fe_2_O_3_ interface, a mechanistic advantage exclusive to these Fe-doped Bi_2_WO_6_ hierarchical microspheres.^67^

Consequently, 20 mM was determined to be the optimal H_2_O_2_ concentration, rather than 30 mM. The systematic optimisation of operational factors determined the following parameters for maximal photocatalytic PNP elimination using 1% Fe_2_O_3_/Bi_2_WO_6_: catalyst dosage of 250 mg L^−1^, pH 7, initial concentration (*C*_0_) of 20 mg L^−1^, and optional H_2_O_2_ concentration of 20 mM. These findings collectively have substantial implications for sustainability. Maintaining a neutral pH obviates the necessity for supplementary acid or alkali dosing to condition the influent, thereby diminishing chemical usage and minimizing the production of secondary salts from neutralization in the treated stream.^68^ Optimal performance occurs at a moderate catalyst loading of 250 mg L^−1^, after which efficiency diminishes, indicating that excessive catalyst application is unproductive and wasteful, aligning with the principles of green chemistry that advocate minimal material use. The self-sustaining ·OH production capability of the Fe_2_O_3_/Bi_2_WO_6_ hierarchical microspheres obviates the need for external oxidants like H_2_O_2_ during standard operation, thus avoiding additional chemical input and its related handling, storage, and disposal expenses. The pseudo-first-order kinetic model was validated for all operational parameter settings, offering a reliable and scalable design equation for reactor engineering. Collectively, our findings demonstrate that the 1% Fe_2_O_3_/Bi_2_WO_6_ system works well under mild, reagent-scarce conditions: an essential prerequisite for sustained advanced oxidation in practical wastewater treatment.

#### 3.2.6. Detection of active species

**Fig. 5:**
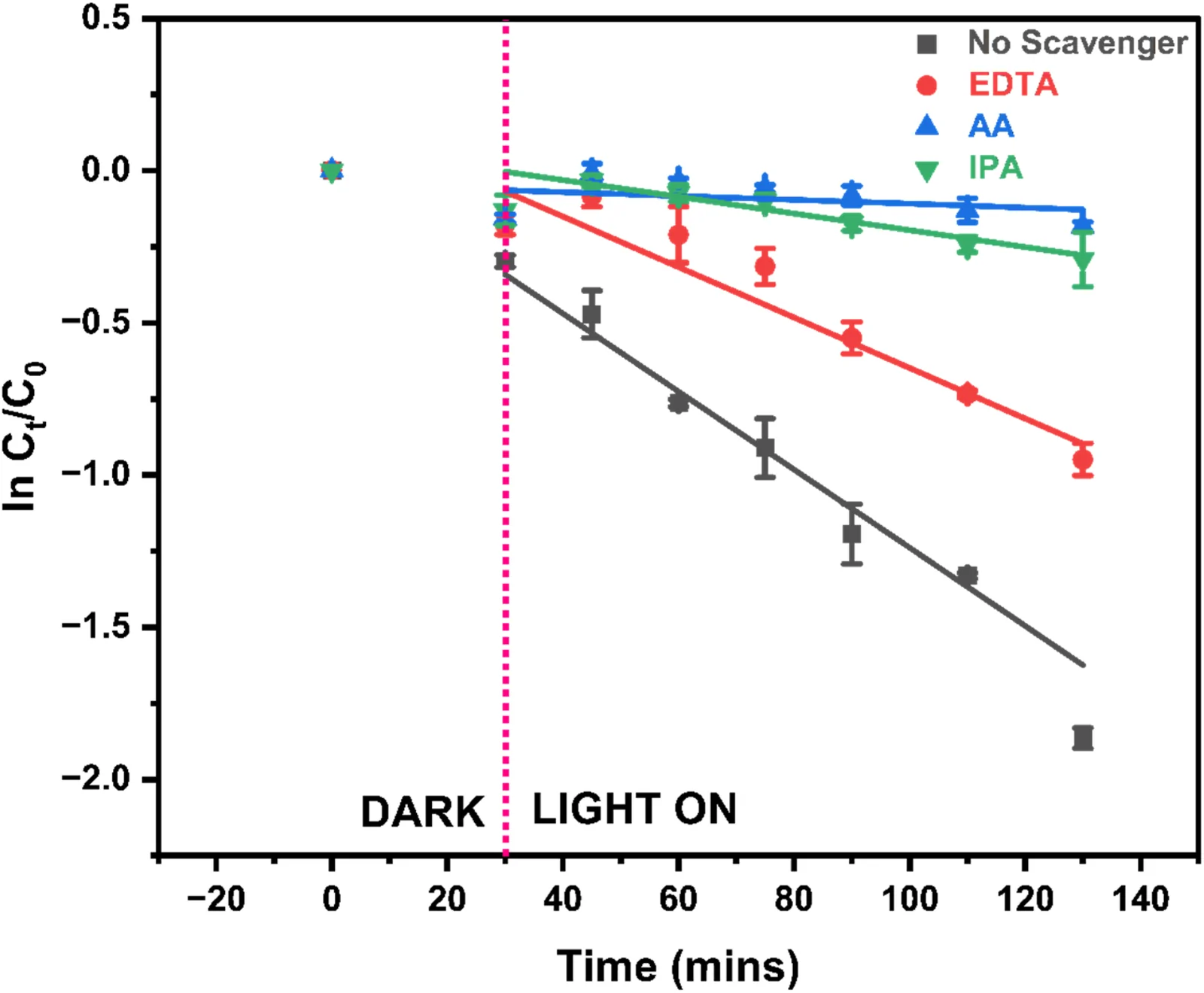
Effect of various scavengers on the degradation of PNP.

To investigate the primary reactive species accountable for PNP degradation on Fe_2_O_3_/Bi_2_WO_6_, radical scavenger tests were conducted under the optimized reaction conditions.^72^ Isopropanol (IPA) was used as a hydroxyl radical (·OH) scavenger, ascorbic acid (AA) as a superoxide radical (·O_2_^−^) scavenger, and EDTA as a hole (h^+^) scavenger, adhering to conventional photocatalytic methodologies. The time-dependent progression of ln (*C*_*t*_/*C*_0_) with and without scavengers (Fig. 5) was modeled using a pseudo-first-order approach to obtain the apparent rate constants *k*_app_. The radical quenching data were examined under the assumption of pseudo-first-order kinetics, with the slope of ln (*C*_*t*_/*C*_0_) plotted against time considered as *k*_app_. In the absence of scavengers, *k*_app_ was 0.0123 min^−1^, which was reduced to 0.0102 min^−1^ with EDTA (hole scavenger), 0.0032 min^−1^ with IPA (·OH scavenger), and 0.0021 min^−1^ with AA (·O_2_^−^ scavenger). Consequently, the degradation rate is suppressed by approximately 17% (EDTA), 74% (IPA), and 83% (AA) compared to the unquenched system, suggesting that superoxide and hydroxyl radicals contribute significantly to PNP oxidation, whereas valence-band holes serve a minor, supportive function.

#### 3.2.7. Catalyst reusability and stability

**Fig. 6:**
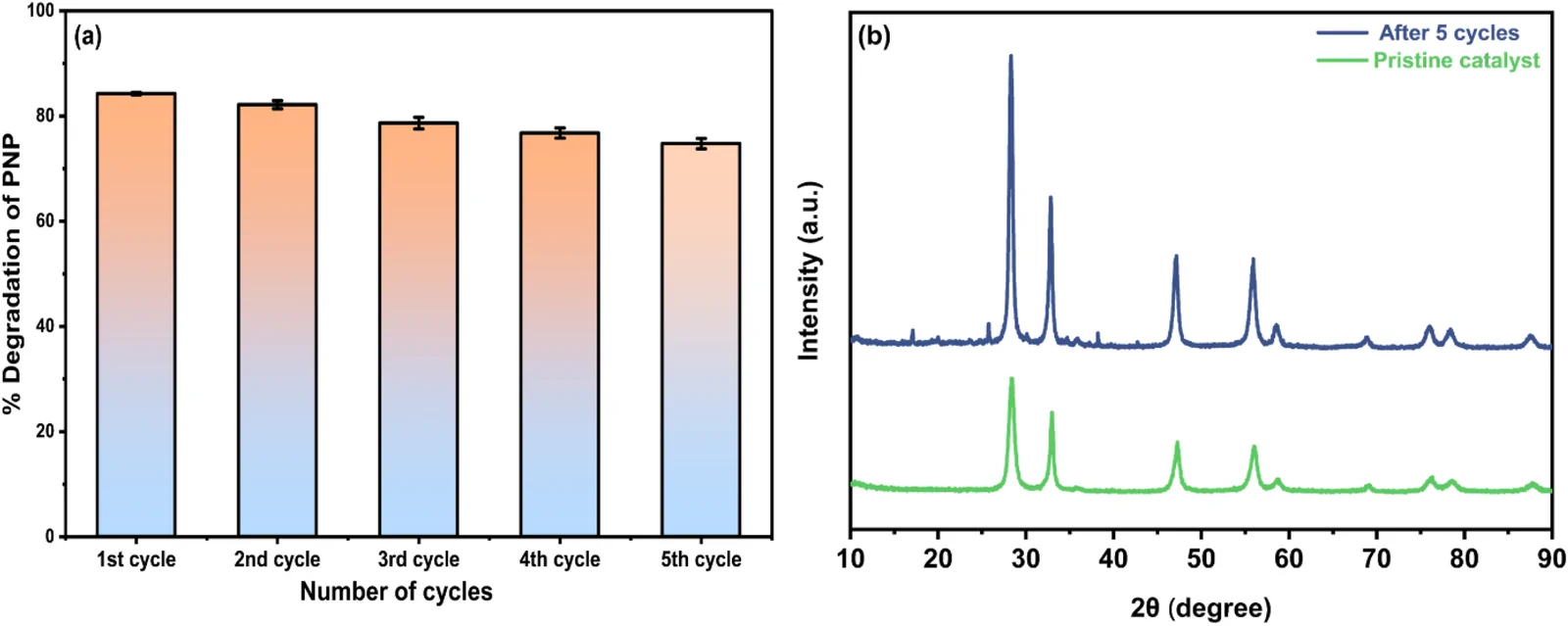
(a) Reusability of 1% Fe_2_O_3_/Bi_2_WO_6_ for PNP degradation over five consecutive cycles under identical experimental conditions. (b) Powder X-ray diffraction patterns of the pristine catalyst and the catalyst recovered after five photocatalytic degradation cycles.

Persistent stability and recyclability of photocatalysts are essential for their effective implementation and economic viability. To study the reusability, the 1% Fe_2_O_3_/Bi_2_WO_6_ composite was extracted from the reaction mixture by centrifugation, thereafter washed with deionized water and ethanol, dried, and heated in a muffle furnace for 4 h at 350 °C. Thereafter, the obtained photocatalyst was employed again in consecutive degradation cycles. Upon continuous exposure to visible light, the photocatalytic efficacy exhibited only a slight reduction over five cycles, suggesting that the Fe-doped Bi_2_WO_6_ hierarchical microspheres preserve most of their functionality and structural stability after reuse. After the fifth cycle, the degradation efficiency of PNP decreased from 84.3% in the first cycle to 74.6% (Fig. 6a). This ∼11.5% drop was due to material losses throughout the recovery process, although the catalyst preserved substantial activity. With no discernible extra crystalline phases or significant peak shifts, the XRD pattern of the catalyst recovered after five photocatalytic cycles maintained the distinctive reflections of the new material, suggesting preservation of the bulk crystal structure within the XRD detection limit.

The post-reaction solutions were analyzed *via* ICP-OES to quantify Fe dissolution during repeated photocatalytic treatment. After the first photocatalytic cycle, the dissolved Fe content was 1.75 µg L^−1^, and after the fifth reuse–regeneration cycle it reduced to 0.863 µg L^−1^, indicating a 50.7% reduction. The low and constant Fe values imply negligible Fe leaching to the aqueous phase and strong chemical stability of the synthesised catalyst after the treatment process after 5 cycles. The ICP-OES results and the unaltered XRD profile (Fig. 6b) of the retrieved catalyst demonstrate that the system functions chiefly as a stable heterogeneous photocatalyst instead of a homogeneous photo-Fenton system.^69^

#### 3.2.8. Mineralization (TOC removal), photocatalytic degradation intermediates and proposed degradation pathway

The basic aim of photocatalytic procedures is to mineralize the specified organic contaminant. Consequently, after the application of the synthesized composites, TOC analysis was employed to evaluate the extent of PNP mineralization. To calculate TOC, eqn (3) was applied as follows:

3 TOC removal (%) = (TOC_0_ − TOC_*t*_)/TOC_0_ × 100,

where TOC_0_ and TOC_t_ are the initial and residual TOC concentrations at time *t* (mg L^−1^), respectively. TOC analysis performed after photocatalytic treatment under the optimised conditions showed 46.7% TOC removal, confirming that the degradation process involved appreciable mineralisation rather than solely the transformation of PNP into intermediate products.

**Scheme 1:**
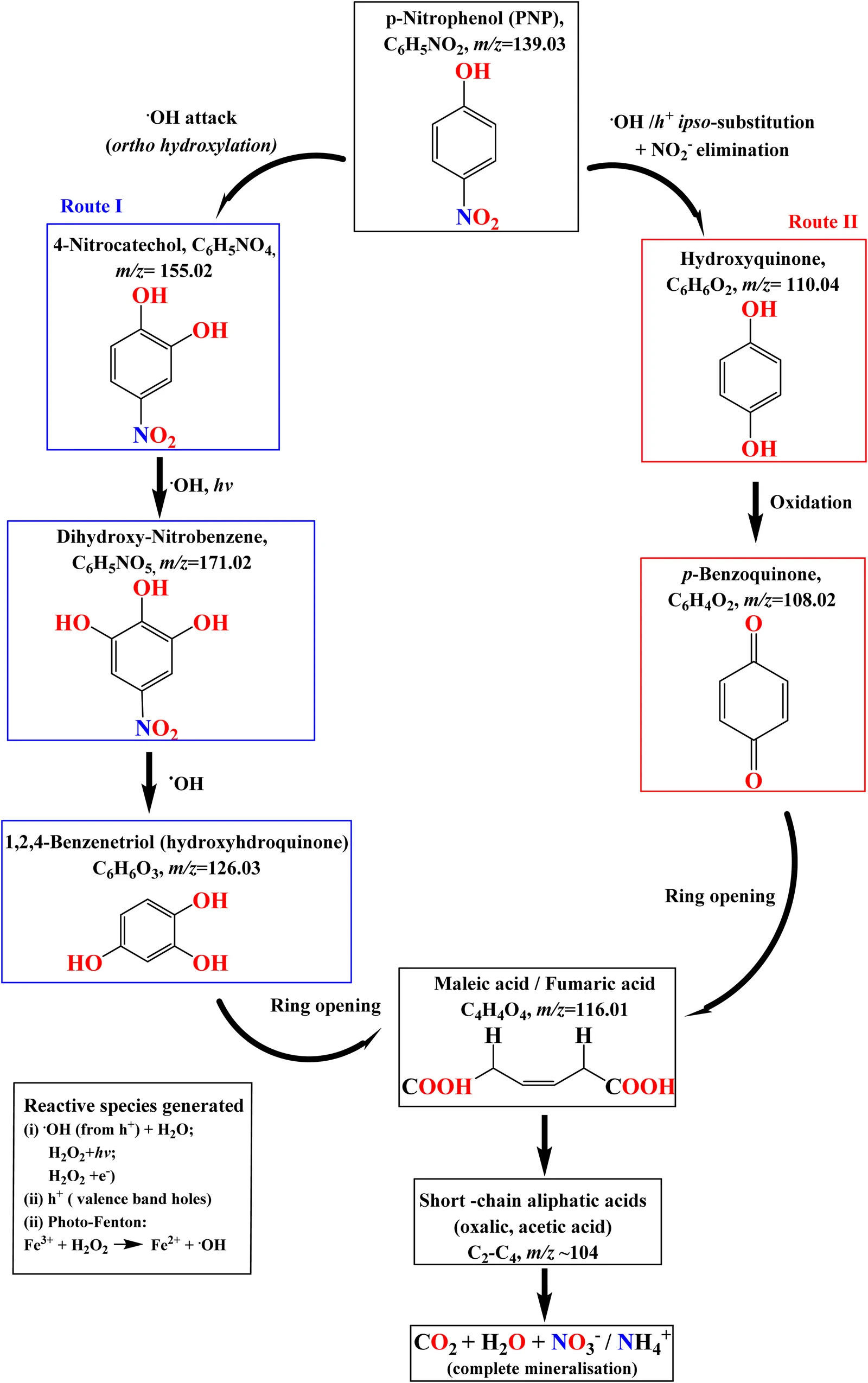
Proposed photocatalytic degradation route of PNP utilising 1% Fe_2_O_3_/Bi_2_WO_6_ in the presence of H_2_O_2_ under visible-light irradiation. Route I: consecutive ·OH-induced hydroxylation resulting in ring cleavage through 1,2,4-benzenetriol. Route II: ipso-substitution with the removal of the nitro group, resulting in hydroquinone and *p*-benzoquinone. Both pathways converge at aliphatic acid intermediates and culminate in full mineralisation. Intermediate assignments utilise HRMS data (ESI^+^, QTOF; *m*/*z* values specified).

To elaborate on the degradation mechanism of PNP using 1% Fe_2_O_3_/Bi_2_WO_6_ under visible-light irradiation, reaction aliquots were investigated using high-resolution mass spectrometry (HRMS, ESI^+^, Waters XEVO G2-XS QTOF). Eight intermediates were found (Table 2), and a two-route degradation strategy is proposed in Scheme 1.

**Table 2:** Proposed degradation intermediates identified during the photocatalytic degradation of *p*-nitrophenol^a^

S. No.	Peak (RRT)	Name (formula)	Compound structure	*m/z* observed [M + H]^+^	Route
1	1.00	*p*-Nitrophenol (C_6_H_5_NO_3_)	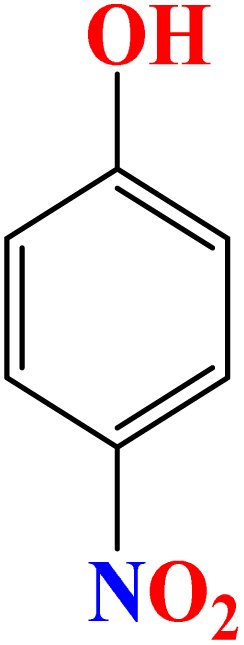	136.07	Parent compound
2	1.78	4-Nitrocatechol (C_6_H_5_NO_4_)	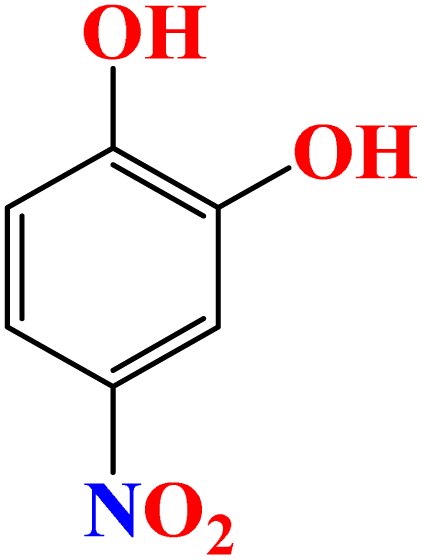	144.04	I
3	1.96	Dihydroxy-nitrobenzene (C_6_H_5_NO_5_)	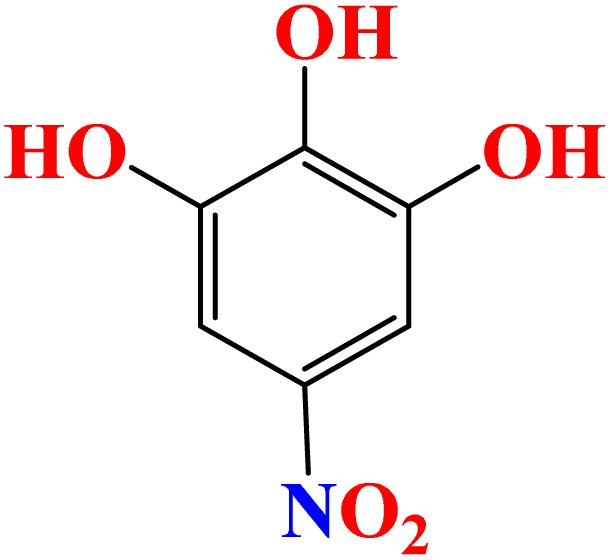	158.02	I
4	0.90	1,2,4-Benzenetriol (C_6_H_6_O_3_)	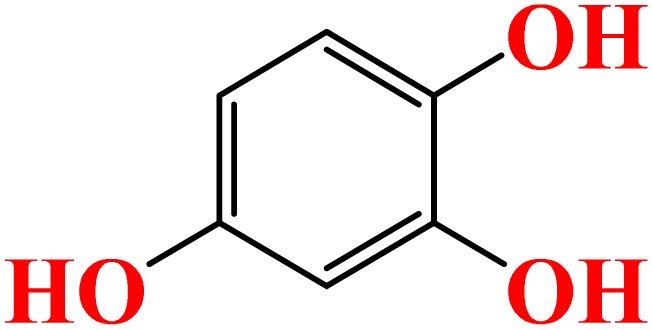	144.04	I
5	0.82	Hydroquinone (C_6_H_6_O_2_)	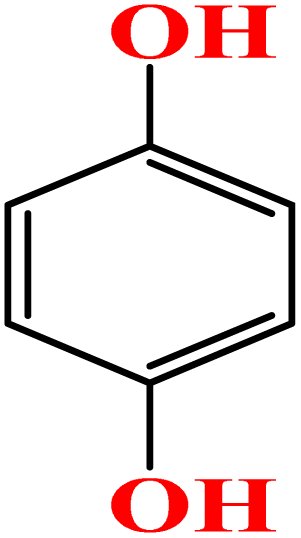	122.06	II
6	0.82	*p*-Benzoquinone (C_6_H_4_O_2_)	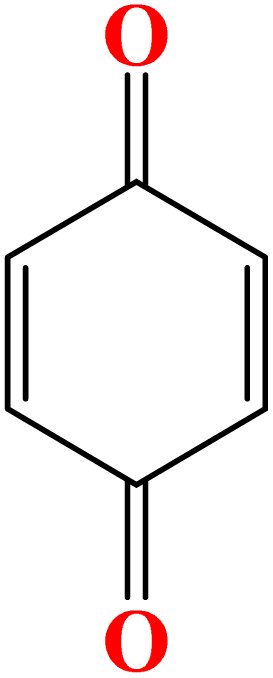	122.06	II
7	1.00	Maleic acid (C_4_H_4_O_4_)	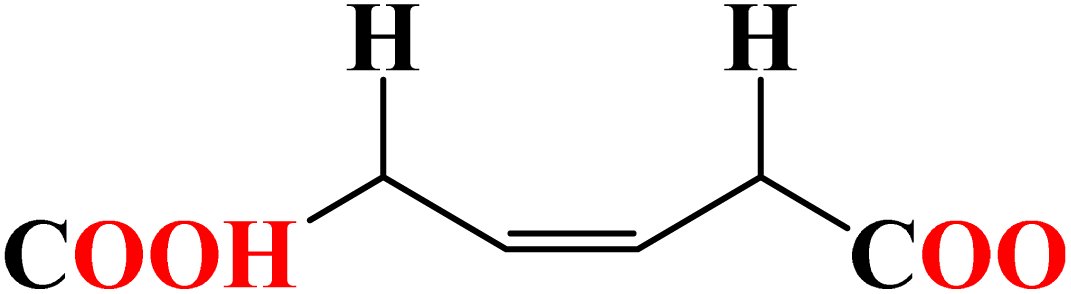	130.03	Both
8	0.79	Short-chain aliphatic acids (*C*_*m*_–*C*_*n*_) *m* and *n* = integers	C_2_–C_4_	104.08	Both

a RRT values are relative to *p*-nitrophenol (RT = 0.796 min) on the XEVO G2-XS QTOF instrument.

The parent compound PNP (C_6_H_5_NO_3_, *m*/*z* = 136.07, [M–OH + H]^+^) undergoes degradation through an enhanced reactive oxygen species (ROS) environment facilitated by three simultaneous mechanisms: photogenerated h^+^/e^−^ at the Fe_2_O_3_/Bi_2_WO_6_ Fe-doped Bi_2_WO_6_ hierarchical microspheres, photolytic cleavage of H_2_O_2_ (H_2_O_2_ + *hv* → 2˙OH), and photo-Fenton cycling of Fe^3+^/Fe^2+^ from Fe_2_O_3_.^70^

In Route I (hydroxylation), sequential ·OH attacks produce 4-nitrocatechol (*m*/*z* = 144.04) → dihydroxy-nitrobenzene (*m*/*z* = 158.05) → 1,2,4-benzenetriol (*m*/*z* = 144.04), culminating in oxidative ring cleavage to maleic acid (*m*/*z* = 130.03, [M + H]^+^).^71^ In Route II (denitrogenation), ipso-substitution of the nitro group yields hydroquinone (*m*/*z* = 122.06) → *p*-benzoquinone (*m*/*z* = 122.06) → ring-opened acids. Both pathways converge at maleic acid, which is then oxidized to short-chain aliphatic acids (*m*/*z* = 104.08) and ultimately mineralized to CO_2_, H_2_O, and NO_3_^−^/NH_4_^+^. Continuous loss of high-*m*/*z* aromatic intermediates and the simultaneous development of low-molecular-weight aliphatic fragments in the HRMS spectra jointly validate the ongoing mineralization over extended irradiation.^72^

#### 3.2.9. Key mechanistic equations

H_2_O_2_ increases the production of OH by direct photolysis, conduction band electron capture, and a Fe_2_O_3_-mediated photo-Fenton pathway, all of which increase reactive oxygen species for faster pollutant destruction^73^ (eqn (4)–(6)) as follows:

4 H_2_O_2_ + e_CB_ → ˙OH + OH^−^

5 H_2_O_2_ + *hv* → 2˙OH

6 Fe^3+^ + H_2_O_2_ → Fe^2+^ + HO_2_˙ + H^+^ (photo-Fenton contribution from Fe_2_O_3_

The proposed degradation pathway of PNP over 1% Fe_2_O_3_/Bi_2_WO_6_ accounts for two routes: hydroxylation and denitration. Both routes ultimately converge through aromatic-ring opening, yielding maleic/fumaric acid, short-chain carboxylic acids, and intermediates. This agrees partly with TiO_2_-based PNP degradation studies, whereby hydroquinone and benzoquinone-type intermediates have been reported before their conversion into small carboxylic acids and mineralization. Conversely, a crucial point of divergence occurs during the initial route. Conventional TiO_2_ photocatalysis commonly relies on UV excitation; PNP removal is enhanced by oxygen aeration and, in some reports, by externally added H_2_O_2_, indicating the importance of electron acceptors and hydroxyl-radical generation. In contrast, the Fe_2_O_3_/Bi_2_WO_6_ is visible-light responsive and is proposed to enable improved electron–hole separation at the semiconductor interface. Additionally, its Fe_2_O_3_ component may also promote an additional photo-Fenton-like route through Fe^3+^/Fe^2+^ cycling and reaction of iron species with *in situ* generated H_2_O_2_, thereby enhancing ˙OH production without needing any external oxidant addition. The occurrence of the nitrocatechol/dihydroxy-nitrobenzene route in the present pathway therefore suggests that PNP hydroxylation and denitration can occur concurrently, whereas the cited TiO_2_ studies emphasize hydroquinone/benzoquinone formation.^74^

#### 3.2.10. Sustainability metrics

To ensure that technological advancements are ecologically sustainable, commercially feasible, and socially equitable, a thorough assessment of their sustainability credentials is vital. Key performance indicators in sustainability metrics analysis include water intensity, mass intensity, reaction mass efficiency, the environmental factor (E-factor), and specific energy consumption.^27^ All metrics (Table 3) were computed from a single optimized experiment: 20 mg L^−1^ PNP, 1% Fe_2_O_3_/Bi_2_WO_6_ at 250 mg L^−1^ (50.00 mg), 84% degradation over 120 min using four 20 W visible-light LEDs (80 W total, 0.16 kWh).

**Table 3:** Green chemistry sustainability metrics for the 1% Fe_2_O_3_/Bi_2_WO_6_ photocatalytic PNP degradation system

Green chemistry metric	Symbol	Value
Mass intensity	MI	16.07 kg kg^−1^
Water/solvent intensity	SI	59 524 kg kg^−1^
Reaction mass efficiency	RME	6.22%
Environment factor	E-factor	15.07 kg kg^−1^
Specific energy consumption	M	∼47 619 kW h kg^−1^

Both the specific electrical energy consumption (47 619 kWh kg^−1^ PNP degraded) and the calculated water intensity (59 524 kg water kg^−1^ PNP degraded) are high in absolute terms and need to be interpreted critically. The experimental scale and dilute reaction conditions are the main causes of these high normalized values: only 3.36 mg of PNP was degraded from 200 mL of a 20 mg L^−1^ PNP model solution. The resulting mass- and energy-intensity values become disproportionately higher when the use of water and electrical input are normalized to such a small amount of eliminated pollutant. Additionally, the experiment was carried out in batch mode for 135 minutes utilizing 4 visible-light LEDs with a nominal total output of 80 W. Therefore, these figures cannot be readily extrapolated to pilot- or industrial-scale applications.

The comparison with other visible-light photocatalytic systems is difficult, and one should be cautious because energy demand is strongly influenced by the light source and wavelength, actual electrical power input, photon flux/irradiance, reactor geometry, treated volume, catalyst loading and configuration, type of pollutant and concentration, water matrix, and target removal efficiency. Photocatalytic reactor scale-up remains challenging because light penetration, mass transfer, catalyst recovery, and additional pumping requirements can affect energy efficiency substantially.

Collectively, these metrics demonstrate that the 1% Fe_2_O_3_/Bi_2_WO_6_ photocatalytic system possesses favorable sustainability characteristics for a laboratory-scale advanced oxidation process. The process employs water as the exclusive solvent, utilizes low-power visible LED irradiation, and generates no secondary hazardous waste streams beyond a recyclable solid catalyst. Even though the absolute values of MI, SI, and M are typical of diluted bench-scale systems, where the low pollutant loading and reaction volume naturally inflate mass and energy-intensity figures, these metrics are not a sign of underlying process inefficiencies and are anticipated to significantly improve upon scaling up to pilot or industrial configurations with higher influent concentrations, continuous-flow operation, and catalyst immobilisation. The absence of corrosive co-reagents, the non-toxic nature of the catalyst constituents (Bi, W, and Fe; no noble metals or persistent organic components), the demonstrated multi-cycle reusability, and the use of energy-efficient visible-light LEDs all support the photocatalytic route's environmental acceptability and scalability as a sustainable strategy for the mitigation of resistant phenolic pollutants in industrial wastewater treatment applications.

With the optimum parameters (pH 7, 250 mg L^−1^ catalyst, 20 mg L^−1^ PNP), the 1% Fe_2_O_3_/Bi_2_WO_6_ composite attained 84.5% reduction in PNP concentration, exhibiting an apparent pseudo-first-order rate constant of *k*_app_ = 0.0093 min^−1^. This performance is analogous to that of recently documented Bi_2_WO_6_-based photocatalysts under visible-light exposure: calcined Bi_2_WO_6_ (BWO400) exhibits *k*_app_ = 0.031 and 0.0209 min^−1^ for RhB and MO with 97% and 92% removal in 120 minutes, respectively; 450-Bi_2_WO_6_ attains *k*_app_ = 0.0261–0.0289 min^−1^ with 85–88% fluoroquinolone elimination in 75 minutes; and Bi_2_WO_6_/SnS_2_ Z-scheme heterostructures indicate *k*_app_ = 0.0082–0.0159 min^−1^ with 91–97% RhB/Cr(vi) removal. While certain heterostructures exhibit elevated rate constants for dyes and antibiotics, the 1% Fe_2_O_3_/Bi_2_WO_6_ catalyst functions within the same kinetic framework, addressing a more resistant nitro-aromatic contaminant, highlighting its competitiveness within contemporary visible-light Bi_2_WO_6_-based systems.^75^

#### 3.2.11. Treatment of a PNP-spiked groundwater sample

To assess the practical applicability of the synthesised photocatalyst, 1% Fe_2_O_3_/Bi_2_WO_6_ hierarchical microspheres, for PNP abatement in a complex water matrix, photocatalytic experiments were performed using a real groundwater sample collected from the northern part of India. The collected water was spiked with PNP to obtain an initial concentration of 20 mg L^−1^. The groundwater exhibited the following physicochemical characteristics: pH 7.7, total hardness = 193 mg L^−1^, bicarbonate and carbonate concentrations of 123 and 70 mg L^−1^, respectively, and a chemical oxygen demand (COD) of 58 mg L^−1^ and BOD_5_ of 25.6 mg L^−1^ O_2_. The concentrations of Cu, Fe, and Mn were 0.314, 0.112, and 0.338 mg L^−1^, respectively. The visible-light-driven photocatalytic degradation experiment was carried out under the optimal operating conditions established using Milli-Q water. As seen in Fig. S6, the initial drop in the PNP concentration in the spiked groundwater was around 3.2% over the 30 min dark-equilibration, showing a small adsorption of the PNP on the catalyst surface. Under visible-light illumination, the concentration of PNP gradually reduced and achieved 69.1% degradation after irradiation for 100 min (total treatment period of 130 min). Kinetic profiles comparison showed that the PNP degradation in spiked groundwater was lower than the synthesized PNP solution under the same optimal conditions. This reduction may arise from the complex constitution of groundwater, where bicarbonate/carbonate species, naturally occurring dissolved organic matter (partly represented by the initial COD), and coexisting metal ions could compete with reactive oxygen species, absorb incident light, or occupy active sites on the photocatalyst surface. Nevertheless, the high PNP removal obtained in the spiked groundwater suggests that the photocatalyst still shows a remarkable activity in a real-water matrix and supports its promise for practical applications for visible-light-assisted water treatment.

## 4. Conclusion

A simple one-step hydrothermal technique was employed to synthesize Fe_2_O_3_/Bi_2_WO_6_ Fe-doped Bi_2_WO_6_ hierarchical microsphere composites. In addition to exhibiting Fe-induced structural modifications, hierarchical nanosheet-based morphology, band-gap narrowing from 3.1 to 2.9 eV, and enhanced charge-carrier separation, the optimized composite retained the orthorhombic Bi_2_WO_4_ structure. The catalyst demonstrated significant PNP transformation and partial mineralization under ideal neutral-pH conditions, achieving 84.5% PNP degradation and 46.7% TOC elimination. To assess the photocatalytic process's environmental viability, green chemistry metrics and real wastewater treatment were also studied. The green-metric evaluation revealed substantial water and energy intensities in dilute laboratory-scale batch conditions, despite the catalyst operating under benign circumstances with water as the reaction medium and no noble metals. As a result, rather than being immediately industrially sustainable, the technique should be viewed as promising. All things considered, 1% Fe_2_O_3_/Bi_2_WO_6_ offers a viable noble-metal-free visible-light photocatalyst for the remediation of phenolic pollutants. To establish catalytic robustness and practical applicability in complex wastewater matrices, future studies should optimize real-wastewater parameters, specifically pH, pollutant concentration, catalyst dose, irradiation period, ionic strength, dissolved organic matter, and competing ions. Transformation-product quantification, catalyst stability/leaching evaluation, real-wastewater validation, and scale-up investigations using recoverable catalysts are further research requisites. Green metrics (MI, WSI, E-factor, RME, and specific energy consumption) suggest that, despite elevated mass and energy intensities at the laboratory scale, substantial improvements are anticipated with increased influent concentrations and by catalyst immobilization and reuse. All of these findings point to 1% Fe_2_O_3_/Bi_2_WO_6_ as an effective, sustainable, and noble-metal-free visible-light photocatalyst for the removal of stubborn phenolic pollutants from industrial wastewater.

## Author contributions

Manmeet Kaur: writing the original draft, experimentation, methodology, investigation, and data curation; Amit Dhir: writing–review and editing, validation, supervision, and conceptualization; and Shilpi Verma: writing – review and editing, data validation, supervision, formal analysis, and conceptualization.

## Conflicts of interest

The authors declare no competing financial interests.

## Supplementary Material

RA-OLF-D6RA06864B-s001

## Data Availability

The data supporting the results of this study are available from the corresponding authors upon reasonable request. Supplementary information (SI): Photocatalytic reactor setup (Fig. S1), preliminary studies on the effectiveness of the synthesised photocatalyst (Fig. S2), EDS elemental mapping (Fig. S3), XPS analysis of the 1% Fe_2_O_3_/Bi_2_WO_6_ (Fig. S4), rate constant (*K*) plots (Fig. S5 and S6) PNP degradation kinetics in synthetic and spiked groundwater supporting the discussion in the main text. See DOI: https://doi.org/10.1039/d6ra06864b.
