# Supplementary material for: Fe2O3/Bi2WO6 hierarchical microspheres for visible-light-driven degradation of p-nitrophenol: performance, mechanism, and green metrics
Source: RSC Adv. 2026 Sep 9;16(43):53592–612. doi: 10.1039/d6ra06864b (PMC13555527; doi:10.1039/d6ra06864b)
Supplement: Supplementary file 1 [file RA-016-D6RA06864B-s001.pdf]

Supplementary Information (SI) for RSC Advances.
This journal is © The Royal Society of Chemistry 2026

Supplementary Information

Fe_2O_3/ Bi_2WO_6 hierarchical microspheres for visible-light-driven degradation of p-nitrophenol: performance, mechanism, and green metrics

Manmeet Kaur, Amit Dhir*, Shilpi Verma*

Department of Energy & Environment, Thapar Institute of Engineering & Technology, Patiala- 147004, Punjab, India

*Corresponding author- amit.dhir@thapar.edu; shilpi.verma@thapar.edu

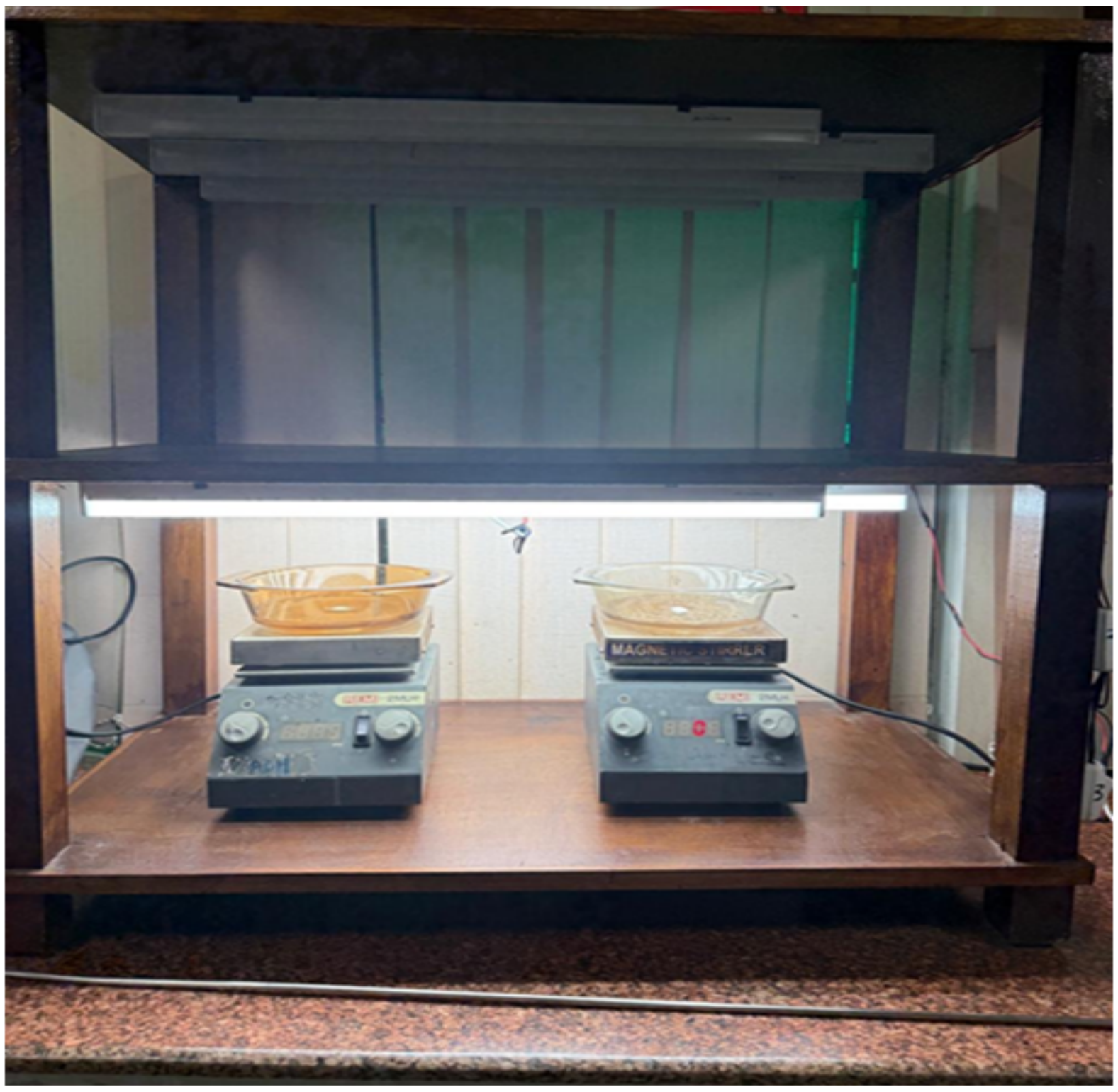

Fig. S1. Laboratory-scale photoreactor for carrying out photocatalytic degradation experiments

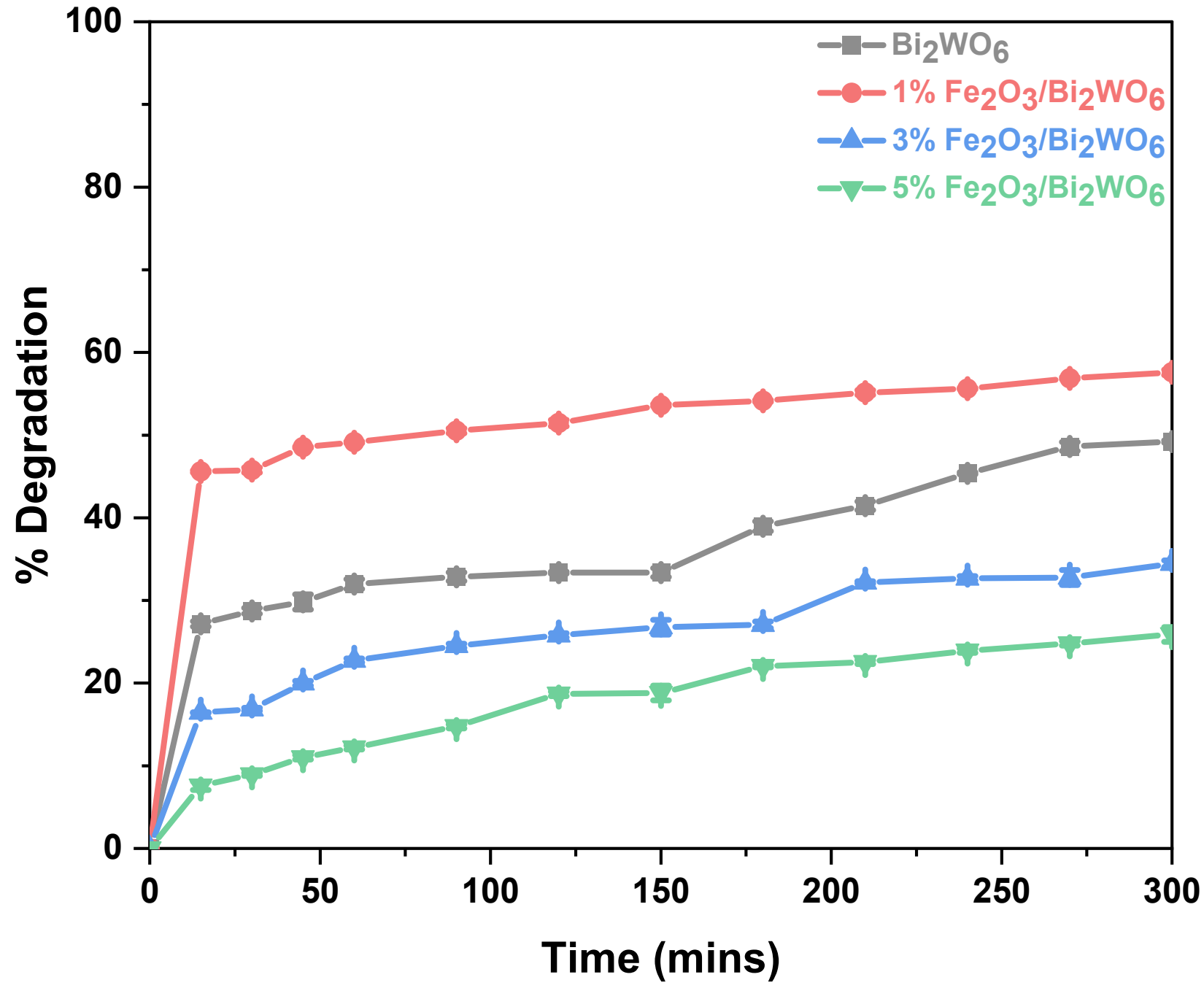

Fig. S2. Initial study of photocatalytic degradation of p-nitrophenol (PNP) over Bi_2WO_6 and Fe_2O_3/Bi_2WO_6 composites (1, 3, and 5 wt%)

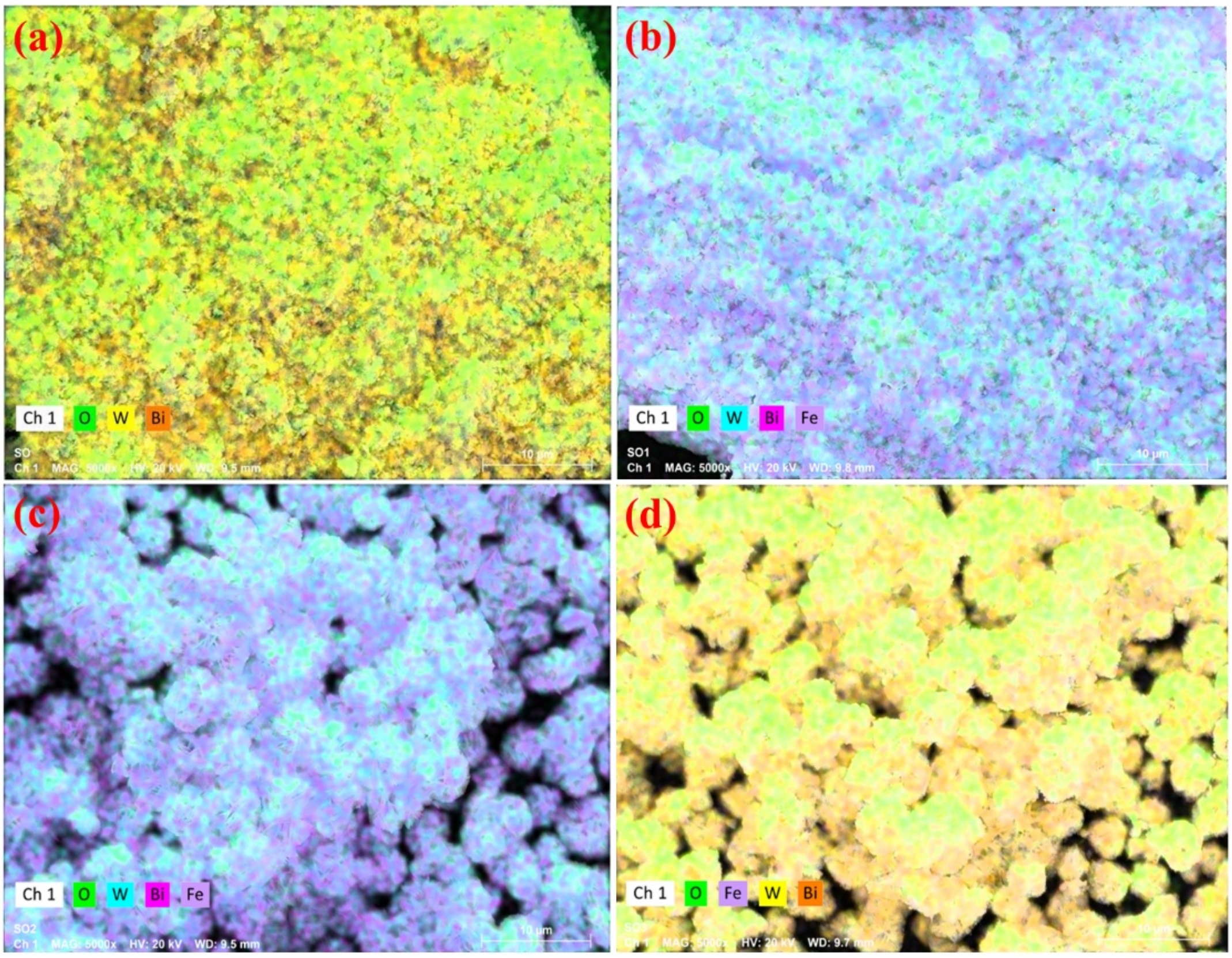

Fig. S3: EDS mapping of (a) $Bi_2WO_{6,}$ (b) 1% Fe_2O_3/Bi_2WO_6; (c) 3% Fe_2O_3/Bi_2WO_6; (d) 5% Fe_2O_3/Bi_2WO_6

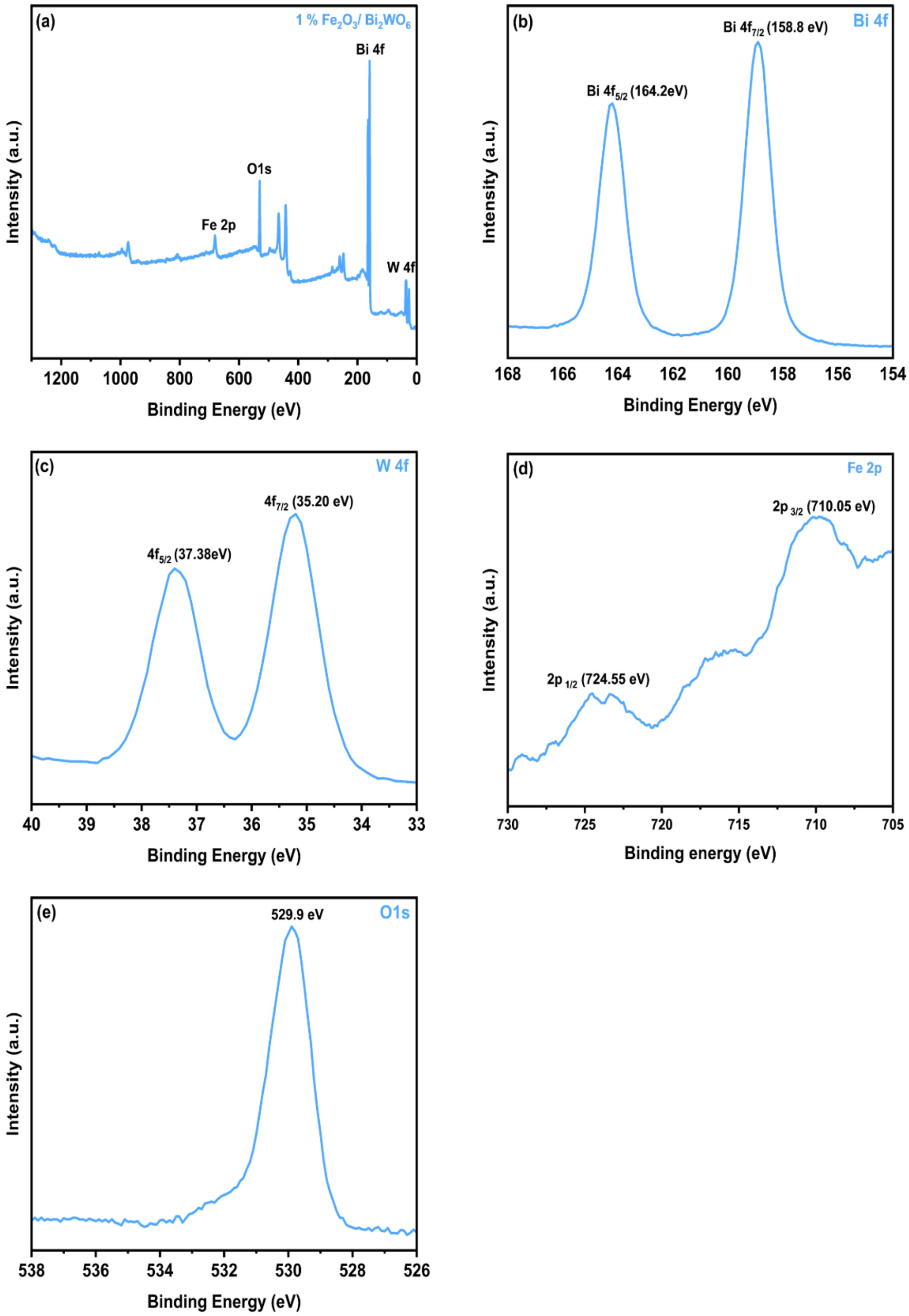

Fig. S4. XPS analysis of the 1% Fe_2O_3/Bi_2WO_6: (a) survey spectrum, (b) Bi 4f (c) W 4f (d) Fe 2p and (e) O 1s core-level spectra.

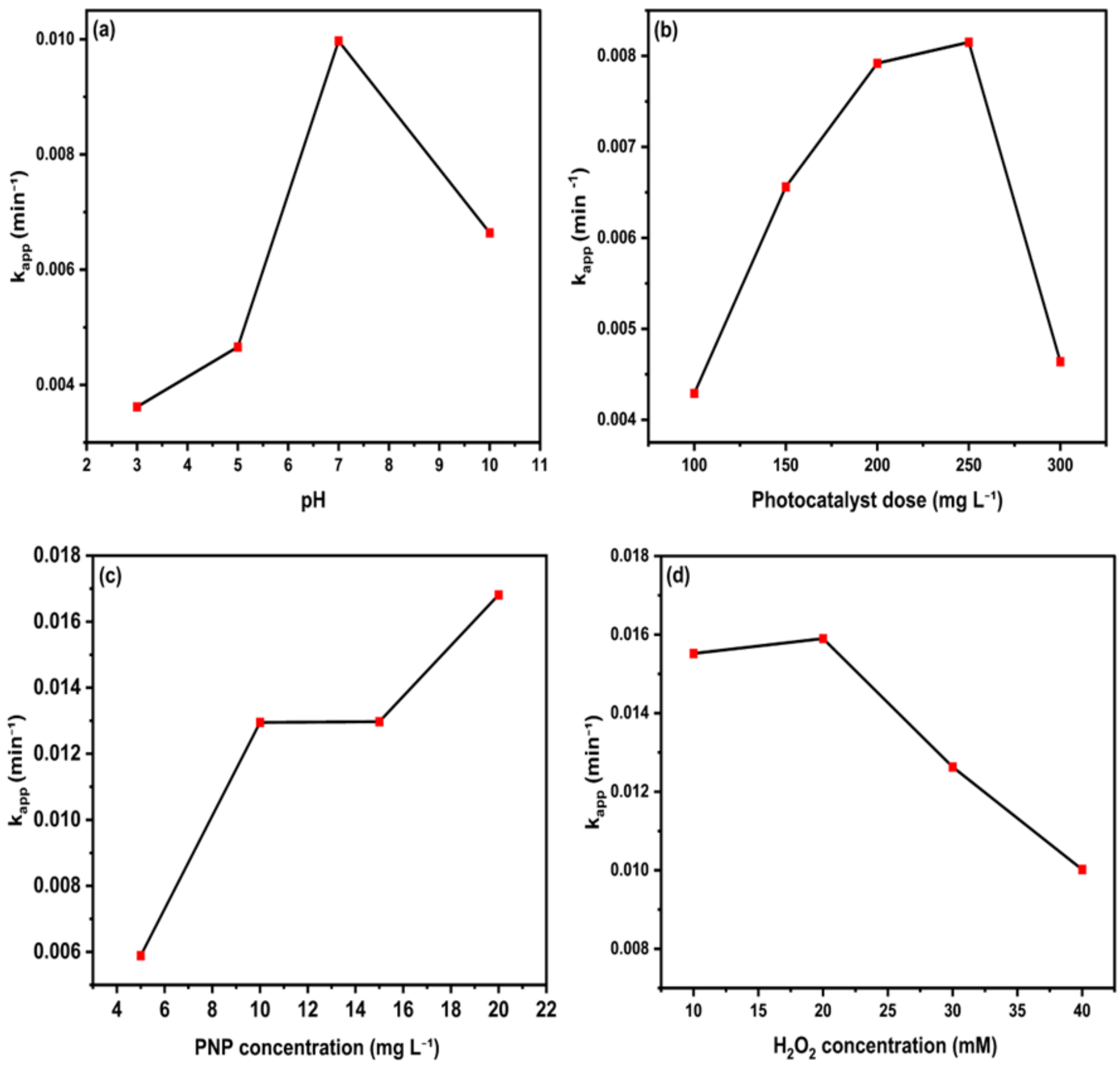

Fig. S5. The rate constant (k) plots under different operating parameters (a) initial pH values of 3, 5, 7 and 10, (b) 1 % Fe_2O_3/ Bi_2WO_6 dosages of 100-300 mg L^{-1}, (c) initial PNP concentrations of 5, 10, 15, and 20 mg L^{-1}, and (d) Influence of H_2O_2 dosages 10-40 mM .

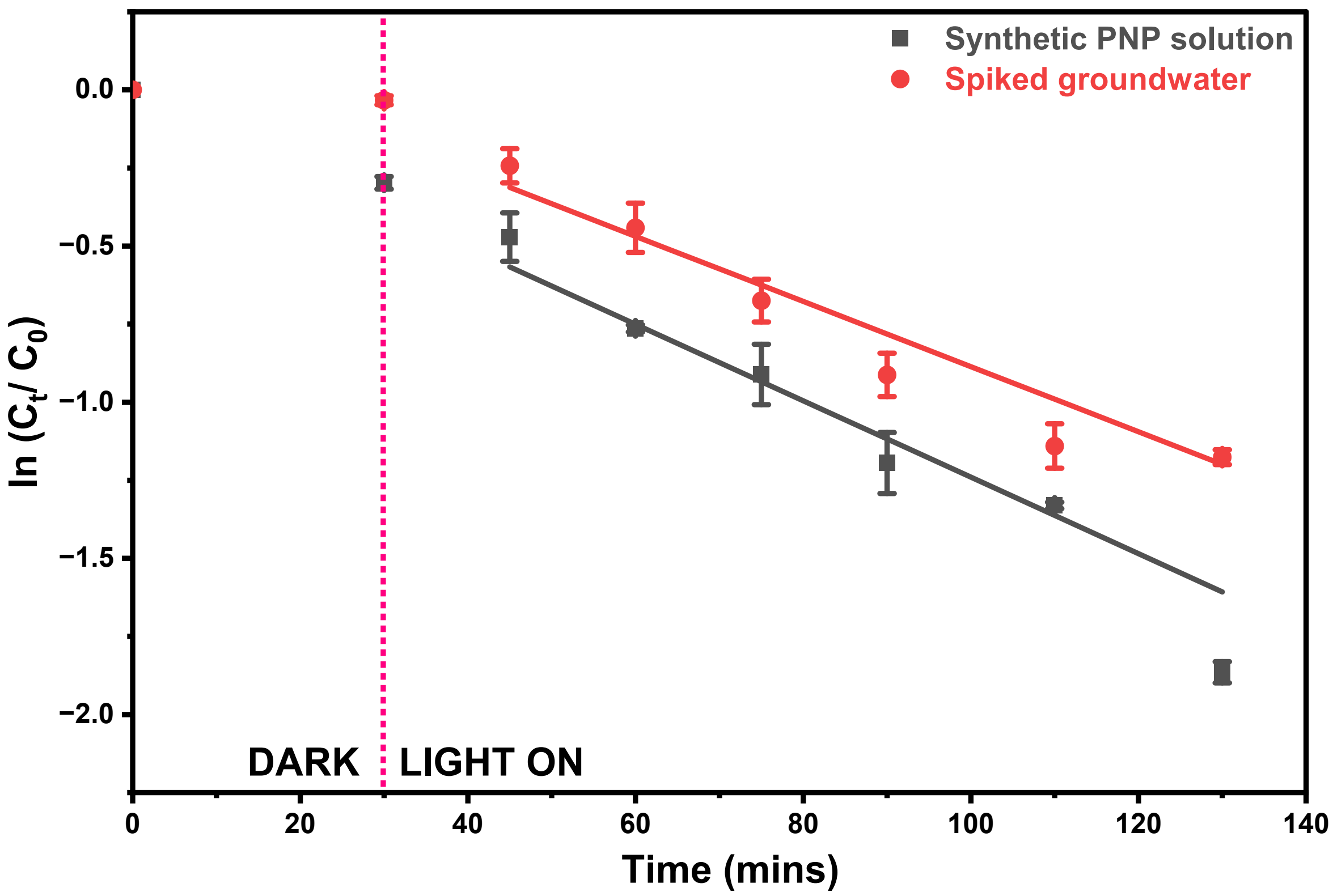

Fig 6. PNP degradation kinetics in synthetic and spiked groundwater
